# Demonstration of a ‘leapfrog’ randomized controlled trial as a method to accelerate the development and optimization of psychological interventions

**DOI:** 10.1017/S0033291722003294

**Published:** 2023-10

**Authors:** Simon E. Blackwell, Felix D. Schönbrodt, Marcella L. Woud, Andre Wannemüller, Büsra Bektas, Max Braun Rodrigues, Josefine Hirdes, Michael Stumpp, Jürgen Margraf

**Affiliations:** 1 Mental Health Research and Treatment Center, Faculty of Psychology, Ruhr-Universität Bochum, Bochum, Germany; 2 Department of Psychology, Ludwig-Maximilians-Universität München, Munich, Germany

**Keywords:** Adaptive trial, Bayes factor, clinical translation, leapfrog, sequential Bayesian analysis

## Abstract

**Background:**

The scale of the global mental health burden indicates the inadequacy not only of current treatment options, but also the pace of the standard treatment development process. The ‘leapfrog’ trial design is a newly-developed simple Bayesian adaptive trial design with potential to accelerate treatment development. A first leapfrog trial was conducted to provide a demonstration and test feasibility, applying the method to a low-intensity internet-delivered intervention targeting anhedonia.

**Methods:**

At the start of this online, single-blind leapfrog trial, participants self-reporting depression were randomized to an initial control arm comprising four weeks of weekly questionnaires, or one of two versions of a four-week cognitive training intervention, imagery cognitive bias modification (imagery CBM). Intervention arms were compared to control on an ongoing basis via sequential Bayesian analyses, based on a primary outcome of anhedonia at post-intervention. Results were used to eliminate and replace arms, or to promote them to become the control condition based on pre-specified Bayes factor and sample size thresholds. Two further intervention arms (variants of imagery CBM) were added into the trial as it progressed.

**Results:**

*N* = 188 participants were randomized across the five trial arms. The leapfrog methodology was successfully implemented to identify a ‘winning’ version of the imagery CBM, i.e. the version most successful in reducing anhedonia, following sequential elimination of the other arms.

**Conclusions:**

The study demonstrates feasibility of the leapfrog design and provides a foundation for its adoption as a method to accelerate treatment development in mental health. Registration: clinicaltrials.gov, NCT04791137.

Given the scale of the global mental health burden, we need time- and resource-efficient methods for the development, testing, and optimization of psychological interventions. However, this need stands in contrast to the present reality: The standard route for treatment development is slow and inefficient; typically proceeding via initial single case or cohort designs followed by a series of 2- or 3-arm randomized controlled trials (RCTs), this process is essentially the same as it was a few decades ago and is unable to keep pace with the demand for improved outcomes in mental health. In other areas of health, such as cancer, recognition of the need for more rapid treatment development has led to the creation of a new generation of alternative research designs (e.g. Hobbs, Chen, and Lee, 2018; Wason and Trippa, 2014). Building on these methodological innovations, a new research method has recently been proposed to accelerate the development and optimization of psychological interventions in mental health: the ‘leapfrog’ trial (Blackwell, Woud, Margraf, & Schönbrodt, 2019).

The leapfrog design can be considered a simple form of an ‘adaptive platform trial’ (Angus et al., 2019). These trials aim to offer an efficient means to test multiple interventions simultaneously, with interim analyses used to adjust the trial design. For example, in the platform design described by Hobbs et al. (2018), multiple new treatments are tested against a control arm (e.g. an established treatment), with repeated Bayesian analyses based on posterior predictive probability used to rapidly eliminate those treatments judged to have a low chance of reaching a pre-specified success criterion. Other platform designs use Bayesian analyses to make adjustments to the randomization weights for individual arms based on their performance, such that participants are preferentially allocated to better-performing arms and those performing poorly are eventually eliminated (e.g. Wason and Trippa, 2014). The leapfrog design was conceived as a type of adaptive platform trial that would lend itself easily to the kinds of comparisons typical in psychological treatment development (e.g. differences in symptom change between treatments) using a relatively simple analytic method, and that additionally included a mechanism for continuous treatment development and optimization via replacement of the control condition over time (Blackwell et al., 2019).

How does a leapfrog trial work? In such a trial, multiple treatment arms can be tested simultaneously, with one designated as the comparison arm or control. As the trial proceeds and data accumulates, sequential Bayes Factors (BFs; e.g. Schönbrodt, Wagenmakers, Zehetleitner, and Perugini, 2017) are calculated on an ongoing basis, enabling repeated comparison of each arm to the control for the chosen outcome without compromising the statistical inferences. These BFs continuously quantify the strength of evidence for or against superiority of a treatment arm over the control, allowing poorly performing arms to be dropped from the trial as soon as there is sufficient evidence to do so. Further, new treatment arms informed by the latest research findings can be incorporated directly into an ongoing trial. Finally, if sufficient evidence accumulates to suggest a treatment arm is better than the control, the control arm is dropped and replaced by this superior arm. These design features provide a route for accelerated treatment development: they substantially reduce the sample sizes needed, they enable a close link between basic and applied research, reducing the time needed for innovation, and they provide mechanisms for continuous treatment optimization consolidated within one trial infrastructure. However, for any new methodology a crucial step is a first demonstration, to show that the method works in practice and not just on paper, and that the promised advantages are not just theoretical, but are also borne out in reality.

We therefore aimed to conduct a leapfrog trial to provide a concrete demonstration from pre-registration to final reporting, as well as to ascertain the design's feasibility and identify any potential problems or adjustments needed. We applied the design to an internet-delivered cognitive training intervention, imagery cognitive bias modification (imagery CBM; Blackwell et al., 2015). Imagery CBM was originally developed in the context of depression, with negative interpretation biases and deficits in positive mental imagery as the target mechanisms (Everaert, Podina, & Koster, 2017; Holmes, Blackwell, Burnett Heyes, Renner, & Raes, 2016). More recently applications have focused specifically on anhedonia (e.g. Blackwell et al., 2015; Westermann et al., 2021), a challenging clinical target where there is great need for treatment innovation (e.g. Craske, Meuret, Ritz, Treanor, and Dour, 2016; Dunn et al., 2020). Imagery CBM currently stands at a critical stage of clinical translation, needing to make the crucial transition from early-phase trials to tests of real-world implementations. Further, while research suggests promise for reducing anhedonia, it also indicates the need for further development work (Blackwell et al., 2015). Imagery CBM therefore made a particularly suitable exemplar for a leapfrog trial.

In applying the leapfrog design to imagery CBM as a low-intensity intervention to reduce anhedonia, our primary aim in this study was to implement and thus demonstrate the core features of a leapfrog trial: Sequential Bayesian analyses based on Bayes Factors (BF), updated continuously with each new participant completing the trial; removal of a trial arm when it hit a prespecified threshold for failure; introduction of new trial arms into an ongoing trial; and replacement of the control arm with a different arm when that intervention arm reached a pre-specified threshold for success.

## Methods

### Design

The study was a randomized controlled leapfrog trial with multiple parallel arms. Allocation was on an equal ratio between those arms currently in the trial at the time of an individual participant's randomization (e.g. 1:1:1 ratio when there were three arms in the trial; 1:1 ratio when there were two arms in the trial). The trial was prospectively registered on clinicaltrials.gov (NCT04791137) and the Open Science Framework (https://osf.io/6k48m; including full study protocol). The study was conducted entirely in German.

### Participants and recruitment

Participants were recruited primarily online, e.g. via social media posts and paid adverts, with adverts indicating that the study involved an online training program that could help with depressed or low mood (see online Supplementary Methods for details). Adverts directed participants to the study website, where they could provide informed consent and register for the study.

Inclusion criteria were: Aged ≥ 18; scoring ≥ 6 on the Quick Inventory of Depressive Symptomatology (QIDS; Rush et al., 2003), indicating at least mild levels of depression symptoms*^1^; fluent German; willing and able to complete all study procedures (including having a suitable device/internet access); and interested in monitoring mood over the study time-period (one month). The depression criterion was assessed during the baseline assessment; all others were self-verified by the participant via the online consent form. There were no exclusion criteria. No financial or other incentives were offered for participation.

The study was conducted entirely online. Study procedures (assessments, interventions, participant tracking) were implemented via a web-based platform and were entirely automated and unguided, with contact from researchers only in response to queries from the participants or to provide technical information (e.g. website unavailability).

### Interventions

#### Initial control arm: monitoring

The control arm at study initiation comprised weekly completion of symptom questionnaires (see below for details). This was chosen as the initial control arm primarily to increase the likelihood of another arm reaching BF_success_, thus enabling us to fulfill our aim of implementing replacement of the control arm. To reduce attrition, once participants had completed the post-intervention assessment they could try out one of the other intervention arms.

#### Intervention arms: imagery cognitive bias modification (imagery CBM)

*General overview.* All intervention arms were variants of imagery CBM, adapted from previous experimental (e.g. Holmes, Lang, and Shah, 2009) and clinical (e.g. Blackwell et al., 2015; Westermann et al., 2021) studies. The interventions comprised a series of training sessions (e.g. 5–20 min each, depending on the arm). Training stimuli were brief audio recordings of everyday scenarios, which always started ambiguous but then resolved positively. Participants were instructed to listen to and imagine themselves in the scenarios as they unfolded; via repeated practice imagining positive outcomes for ambiguous scenarios during the training sessions, imagery CBM aims to instill a bias to automatically imagine positive outcomes for ambiguous situations in daily life. Participants also completed weekly questionnaires, as in the monitoring arm. The different intervention variants are here designated version 1 (v1), version 2 (v2) etc. Fuller descriptions are provided in the online Supplementary Methods.

*CBMv1.* CBMv1 started with an initial introductory session comprising an extended introduction to mental imagery followed by 20 training scenarios (as per Westermann et al., 2021). The first two training weeks then included four sessions with 40 scenarios each, and the final two weeks included two sessions of 40 scenarios.

*CBMv2.* CBMv2 had an identical schedule to CBMv1, but a more extended rationale for the training was provided, as well as further instructions and suggestions to recall and rehearse the scenarios in daily life, for example when encountering a similar situation, with the aim of enhancing training transfer (see Blackwell, 2020; Blackwell and Holmes, 2017).

*CBMv3.* CBMv3 tested a schedule of more frequent, briefer training sessions. Following the introductory session, on five days of each training week there were two brief training sessions scheduled for completion (15 scenarios, approx. 5 min). It was planned that if CBMv2 had a higher Bayes Factor *v.* control than CBMv1 when CBMv3 was introduced, the additional instructions from CBMv2 would also be used for CBMv3.

**Fig. 1.**
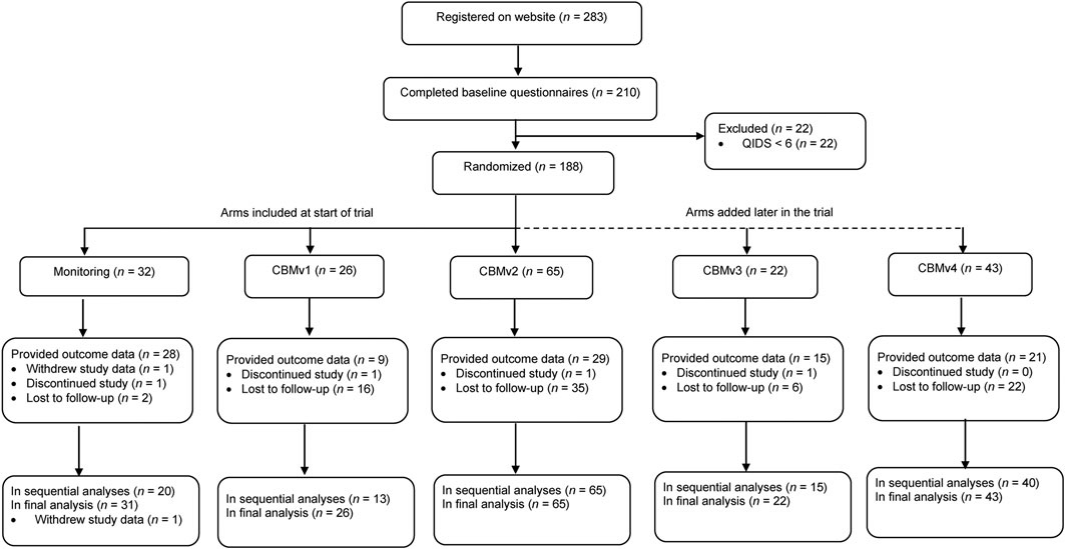
Flow of participants through the study. Note that not all trial arms were included simultaneously in the trial (see Fig. 2).

*CBMv4.* The specifications of CBMv4 were decided upon during the trial, based on data and feedback from participants completing CBMv1 and CBMv2, in particular the higher than expected attrition rates (see Fig. 1 and Table 1). Instead of implementing our original plan for a fourth CBM arm, we designed CBMv4 to increase adherence and acceptability, by including shorter sessions (32 instead of 40 scenarios per session, only 10 in the introductory session), fewer sessions in the first training weeks (2 in the first week, 3 in subsequent weeks), and more engaging task instructions (building on those used in CBMv2) including encouragement to keep training even if participants felt they were not yet benefitting.

**Table 1.** Final outcomes for each training arm *v.* the relevant comparison arm, including all participants randomized

	CBM v1	CBMv2	CBMv3	CBMv4
Comparison arm	Monitoring	Monitoring	CBMv2	CBMv2
*N* for comparison				
This arm	26	29	22	43
Comparison arm	27	31	18	36
% Outcome data				
This arm	34.62%	48.28%	68.18%	48.84%
Comparison arm	92.59%	90.32%	50.00%	41.67%
Post-intervention DARS estimated mean [95% CIs]				
This arm	62.54 [55.14 to 69.95]	72.80 [67.55 to 78.04]	68.62 [63.33 to 73.91]	64.14 [59.30 to 68.99]
Comparison arm	63.98 [59.11 to 68.84]	64.63 [60.66 to 68.59]	69.03 [62.78 to 75.27]	60.97 [55.43 to 66.50]
*d* [95% CIs]	−0.08 [−0.51 to 0.35]	0.55 [0.15 to 0.96]	−0.03 [−0.53 to 0.47]	0.18 [−0.19 to 0.56]
BF	0.176	11.676	0.235	0.502

*Note*: Comparisons between arms include only participants who were concurrently randomized (i.e. who could have been randomized to either arm); hence sample sizes, % outcome data provided and model estimates vary depending on which arms are being compared. Estimated means, CIs, effect size *v.* comparison arm (*d*), and Bayes Factor *v.* comparison arm (BF) derived from the constrained longitudinal data analysis model. DARS, Dimensional Anhedonia Rating Scale. Higher scores on the DARS indicate lower levels of anhedonia. CBM v1/2/3/4, Cognitive Bias Modification version 1/2/3/4.

### Measures

#### Primary outcome

The primary outcome was anhedonia at post-intervention (controlling for baseline scores), measured via the total score on the Dimensional Anhedonia Rating Scale (DARS; Rizvi et al., 2015; Wellan, Daniels, and Walter, 2021). Higher scores indicate lower levels of anhedonia.

#### Secondary outcomes

The QIDS (Roniger, Späth, Schweiger, & Klein, 2015; Rush et al., 2003), GAD-7 (Lowe et al., 2008; Spitzer, Kroenke, Williams, & Lowe, 2006), and Positive Mental Health Scale (PMH; Lukat, Margraf, Lutz, van der Veld, and Becker, 2016) were administered as weekly measures of depression, anxiety, and positive mental health respectively. The Ambiguous Scenarios Test for Depression (AST; Rohrbacher and Reinecke, 2014) and Prospective Imagery Test (PIT; Morina, Deeprose, Pusowski, Schmid, and Holmes, 2011) were administered at baseline and post-intervention as measures of putative mechanisms (interpretation bias and vividness of prospective imagery). The short (20-item) form of the Negative Effects Questionnaire (NEQ; Rozental et al., 2019) was administered at post-intervention to assess self-reported negative effects (see online Supplementary Methods for further information including details of internal consistency).

#### Other measures

The Credibility/Expectancy Questionnaire (CEQ; Devilly and Borkovec, 2000; Riecke, Holzapfel, Rief, and Glombiewski, 2013) was administered at baseline to measure credibility of the intervention rationale and expectancy for symptom improvement. A feedback questionnaire was administered at the end of the study (see online Supplementary Methods for more details).

#### Adverse events

The following Adverse Events were pre-specified: reliable deterioration on the QIDS or GAD-7 from pre- to post-intervention, indicated by an increase greater than a reliable change index calculated from normative data (see online Supplementary Methods); other potential adverse events (e.g. suicidal ideation/acts, self-harm) communicated to the researchers by messages sent by participants during the trial, or via the NEQ.

### Procedure

After registering and logging in to the study platform, participants provided demographic and background clinical information, then completed a sound test to check device compatibility. Next they completed the DARS, QIDS, GAD-7, PMH, AST, SUIS, and PIT, with the baseline QIDS used to determine eligibility. After confirming readiness to start the next phase, participants were randomized to condition (see below). Participants then read a brief description of their assigned condition and completed the CEQ.

Over the next four weeks, participants were scheduled to complete a set of questionnaires (QIDS, GAD-7, PMH) each week, as well as training sessions according to their allocated condition. Automated emails were sent to remind participants about scheduled training sessions or questionnaires, and participants could see a graphical display of their scores within the online system. At four weeks post-baseline, participants were requested to complete the final set of questionnaires, the DARS, QIDS, GAD-7, PMH, AST, and PIT, followed by the NEQ and feedback questionnaire. They then received debriefing information and were given the option of starting a new training schedule of their choice. Participants had one week to complete the final questionnaires, after which they were no longer available and counted as missing data.

### Randomization, allocation concealment, and blinding

Randomization was stratified by gender (female *v.* not female) and baseline score on the QIDS (≤9 *v.* >9, i.e. mild *v.* moderate-to-severe). Randomization was in blocks equal to the number of arms in the trial at the time (e.g. block lengths of 3 when there were 3 arms in the trial). Each time a trial arm was added or removed a new set of randomization sequences were generated, such that participants were only randomized between those arms currently in the trial at that timepoint (see online Supplementary Methods for further details).

Participants were not blind as to whether they were receiving an intervention or monitoring only, but otherwise were blind to the nature of the different versions of the intervention being tested. The research team were not blind to participant allocation, although only had minimal contact with participants (e.g. responding to messages). One researcher (SEB) monitored performance of the arms as data accumulated, via the sequential analyses, and shared outcome data with other researchers (FDS, MLW, AW, JM) for the purpose of decision-making. These researchers were therefore not blind to outcomes as the trial progressed.

### Statistical analyses

All analyses were conducted in RStudio (RStudio, Inc., 2016), running R version 4.1.2 for the analyses in this paper (R Development CoreTeam, 2011).

Sequential analyses, repeated each time new outcome data was provided, were based on computation of directional Bayes factors (BFs) comparing each trial arm individually to the control arm for the primary outcome (DARS at post-intervention, controlling for pre-intervention scores). BFs (e.g. Wagenmakers, 2007) are essentially the probability of the observed data if one hypothesis were true (in this case, the alternative hypothesis that a trial arm was superior to the control arm in reducing anhedonia) divided by the probability of the observed data if another hypothesis were true (in this case, the null hypothesis that a trial arm was not superior to the control arm in reducing anhedonia). Sequential analyses were conducted as intention-to-treat (including all participants randomized to condition). To handle missing data, analyses were conducted via constrained longitudinal data analysis (cLDA; Coffman, Edelman, and Woolson, 2016) using nlme (Pinheiro, Bates, DebRoy, Sarkar, & R Core Team, 2019), and an approximate BF calculated via the t-statistic for the Time × Group effect using BayesFactor (Morey & Rouder, 2015) with a directional default Cauchy prior (*rscale* parameter = √2/2).

As elaborated by Blackwell et al. (2019), how a leapfrog trial proceeds is determined by a pre-specified set of analysis parameters: A minimal sample size per arm, *N*_min_, at which sequential analyses are initiated; a maximum sample size, *N*_max_, at which an arm is removed from the trial (this prevents a trial continuing indefinitely); a BF threshold for failure, BF_fail_, reaching of which results in an arm being removed from the trial; and a BF threshold for success, BF_success_, reaching of which results in an arm becoming the new control arm, with the current control arm being dropped. The specific parameters chosen for any particular trial can be determined by simulation to achieve the desired false-positive and false-negative error rates (at a pairwise or whole trial level) in the context of feasible sample sizes. For this specific trial, as the primary aim was to demonstrate the leapfrog trial features, rather than to draw confident conclusions about the efficacy of the intervention being used as an exemplar, the parameters were chosen to provide lenient thresholds, therefore facilitating this demonstration without requiring large sample sizes. The parameters for this trial were pre-specified as follows: *N*_min_ = 12; *N*_max_ = 40; BF_fail_ = 1/3; and BF_success_ = 3. Simulations with up to 25% missing data suggested that these parameters provided a pairwise false-positive rate of 9% and power of ~25%, ~55%, and ~85% respectively for small (*d* = 0.2), medium (*d* = 0.5) and large (*d* = 0.8) between-group effect sizes (see the protocol and associated simulation scripts at https://osf.io/8mxda/ for full details). When an arm was dropped from the trial, any participants still completing the trial in that arm finished their intervention as usual. Their data did not inform further sequential analyses but was used in calculating the final BF and effect sizes *v.* the relevant control arm.

Effect sizes (approximate Cohen's *d*, including an adjustment for sample size) and estimated means for the primary outcome, as well as their 95% confidence intervals (CIs), were derived from the cLDA model using the packages effectsize (Ben-Shachar, Lüdecke, & Makowski, 2020) and emmeans (Lenth, 2022). Data and analysis scripts can be found at https://osf.io/8mxda/.

## Results

A total of 188 participants were randomized between 14 April 2021 and 12 December 2021, with the last outcome data provided on 12 January 2022. Participant characteristics are shown in Table 2, with more detailed information in online Supplementary Table S1 in the Supplementary Results. Figure 1 shows the flow of participants through the study. While adherence rates were high in the initial control arm, drop-out was higher across the training arms (see Fig. 1 and Table 1).

**Fig. 2.**
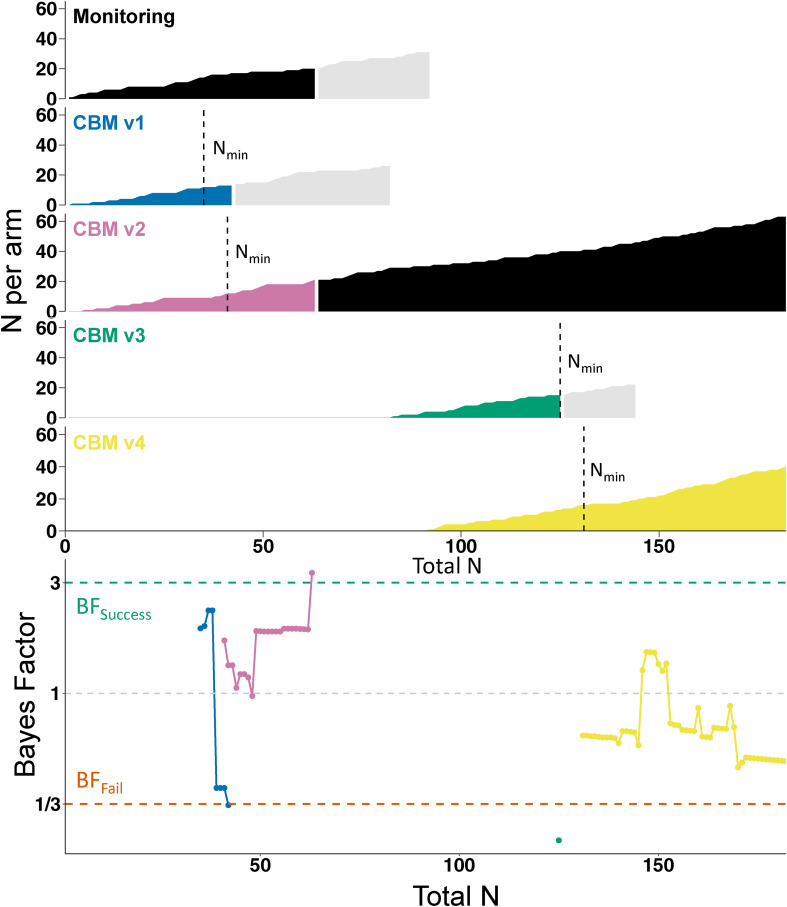
Course of the trial. The top part of the figure shows the accumulation of participant data in each individual arm, plotted against the total number of participants who had completed the trial. Vertical dashed lines indicate when the arm reached the minimum sample size (N_min_) for starting sequential analyses. Black shading indicates the control condition (i.e. initially Monitoring, later CBM v2). Light gray shading indicates participants who had been randomized to an arm but had not yet completed the trial when their allocated arm was dropped; they completed the trial as normal but their data was not used in the sequential analyses. However, their data did inform the final analyses as presented in Table 1. The lower part of the figure shows how the Bayes Factors developed in each arm (identifiable by color).

**Table 2.** Participant characteristics at baseline across trial arms, including all participants randomized

	Monitoring	CBM v1	CBM v2	CBM v3	CBM v4
	*n* = 31	*n* = 26	*n* = 65	*n* = 22	*n* = 43
	*M* (s.d.)/*N* (%)	*M* (s.d.)/*N* (%)	*M* (s.d.)/*N* (%)	*M* (s.d.)/*N* (%)	*M* (s.d.)/*N* (%)
Age (years)	38.94 (13.29)	32.85 (10.22)	41.55 (14.10)	41.18 (10.99)	43.49 (14.22)
Gender					
Male	9 (29.03%)	7 (26.92%)	12 (18.75%)	6 (27.27%)	9 (20.93%)
Female	22 (70.97%)	19 (73.08%)	51 (79.69%)	16 (72.73%)	34 (79.07%)
Diverse/Other	0 (0.00%)	0 (0.00%)	1 (1.56%)	0 (0.00%)	0 (0.00%)
German Nationality	26 (83.87%)	24 (92.31%)	56 (87.50%)	21 (95.45%)	38 (88.37%)
Education level					
No school qualifications	0 (0.00%)	0 (0.00%)	0 (0.00%)	0 (0.00%)	0 (0.00%)
High school diploma or equivalent	11 (35.48%)	12 (46.15%)	24 (37.50%)	9 (40.91%)	17 (39.53%)
Vocational college diploma	9 (29.03%)	3 (11.54%)	9 (14.06%)	5 (22.73%)	10 (23.26%)
Bachelor's degree	2 (6.45%)	6 (23.08%)	9 (14.06%)	5 (22.73%)	7 (16.28%)
Master's degree	8 (25.81%)	3 (11.54%)	19 (29.69%)	2 (9.09%)	6 (13.95%)
Doctorate/Habilitation	1 (3.23%)	2 (7.69%)	3 (4.69%)	1 (4.55%)	3 (6.98%)
Occupation					
Unemployed/seeking employment	1 (3.23%)	3 (11.54%)	7 (10.94%)	1 (4.55%)	3 (6.98%)
Student/trainee	6 (19.35%)	10 (38.46%)	10 (15.62%)	5 (22.73%)	5 (11.63%)
Self-employed	3 (9.68%)	0 (0.00%)	12 (18.75%)	3 (13.64%)	5 (11.63%)
Employed	19 (61.29%)	11 (42.31%)	31 (48.44%)	11 (50.00%)	24 (55.81%)
Retired	2 (6.45%)	2 (7.69%)	4 (6.25%)	2 (9.09%)	6 (13.95%)
Relationship status					
Single	12 (38.71%)	9 (34.62%)	27 (42.19%)	8 (36.36%)	17 (39.53%)
Single, in a stable relationship	5 (16.13%)	4 (15.38%)	12 (18.75%)	4 (18.18%)	9 (20.93%)
Married/Civil partnership/Cohabiting	12 (38.71%)	12 (46.15%)	21 (32.81%)	7 (31.82%)	13 (30.23%)
Divorced/Separated/Widowed	2 (6.45%)	1 (3.85%)	3 (4.69%)	3 (13.64%)	3 (6.98%)
Other	0 (0.00%)	0 (0.00%)	1 (1.56%)	0 (0.00%)	1 (2.33%)
Monthly household income					
up to 899€	6 (19.35%)	8 (30.77%)	7 (10.94%)	6 (27.27%)	6 (13.95%)
900–3099€	17 (54.84%)	9 (34.62%)	44 (68.75%)	8 (36.36%)	20 (46.51%)
3100–5099€	3 (9.68%)	7 (26.92%)	9 (14.06%)	8 (36.36%)	16 (37.21%)
5100€ or more	5 (16.13%)	2 (7.69%)	4 (6.25%)	0 (0.00%)	1 (2.33%)
Current treatment for mental health	7 (22.58%)	7 (26.92%)	16 (25.00%)	2 (9.09%)	9 (20.93%)
Past treatment for mental health	17 (54.84%)	11 (42.31%)	37 (57.81%)	8 (36.36%)	26 (60.47%)
Ever received mental health diagnosis	16 (51.61%)	13 (50.00%)	36 (56.25%)	10 (45.45%)	26 (60.47%)
DARS (baseline)	64.58 (12.48)	59.31 (12.51)	61.69 (14.18)	63.91 (10.18)	58.19 (11.48)
QIDS (baseline)	11.84 (3.64)	12.58 (4.34)	12.43 (3.91)	13.36 (4.15)	13.21 (4.17)
GAD7 (baseline)	10.65 (4.90)	11.31 (4.54)	11.09 (4.59)	11.18 (5.05)	10.58 (4.93)
CEQ Credibility	−0.18 (3.04)	−0.41 (2.38)	0.11 (2.62)	0.55 (2.40)	−0.07 (2.51)
CEQ Expectancy	0.22 (3.04)	−0.63 (2.22)	0.03 (2.66)	−0.18 (2.75)	0.27 (2.60)

CBM v1/2/3/4, Cognitive Bias Modification version 1/2/3/4; DARS, Dimensional Anhedonia Rating Scale; QIDS, Quick Inventory of Depressive Symptomatology; PMH, Positive Mental Health Scale; CEQ, Credibility/Expectancy Scale.

*Note*: A total of 32 participants were randomized into the Monitoring arm, but one withdrew their data from the trial, hence data for only 31 participants are presented here.

### Sequential analyses (primary outcome: anhedonia)

Sequential Bayesian analyses, based on the primary outcome of anhedonia (DARS) at post-intervention, started on 27 May 2021 when both monitoring and CBMv1 hit *N*_min_. Figure 2 shows the progress of the trial and sequential BFs, which were calculated as the trial proceeded and used to make decisions (e.g. removal of an arm) while the trial was ongoing. CBMv1 hit BF_fail_ (BF = 0.3297) once 13 participants had provided outcome data (*v.* 17 in the control condition); CBMv1 was therefore dropped from the trial and CBMv3 was introduced as a new arm. To increase the trial's efficiency, CBMv4 was introduced shortly afterwards^2^. CBMv2 hit BF_success_ (BF = 3.31) after outcome data had been provided for 21 participants (*v.* 20 in the control condition) and therefore became the new control condition, with monitoring (the initial control condition) dropped from the trial. CBMv3 hit BF_fail_ (BF = 0.23) after 15 participants (*v.* 12 participants in CBMv2) and was therefore dropped from the trial. The trial ended with CBMv4 hitting *N*_max_ (*N* = 40, BF = 0.51), at which point CBMv2 was the remaining arm^3^.

### Final outcomes

Table 1 shows final outcomes for each arm *v.* their relevant control, as calculated at the end of the trial including all participants randomized. This indicates that the superiority of CBMv2 over monitoring in reducing anhedonia corresponded to a medium effect size (*d* = 0.55), and a BF providing strong evidence of superiority (BF = 11.676).

### Secondary outcomes

Details of secondary outcomes are provided in the online Supplementary Results.

### Adverse events

No Serious Adverse Events were recorded. We recorded the following Adverse Events: Two participants showed reliable deterioration on the QIDS (one in monitoring, one in CBMv1), and four participants showed reliable deterioration on the GAD7 (two in monitoring, one in CBMv2, one in CBMv3). For further details of negative effects (NEQ) see the online Supplementary Results.

## Discussion

This study provides a successful first demonstration of a ‘leapfrog’ trial applied to the development of a psychological treatment, in this case an internet-delivered cognitive training intervention targeting anhedonia, and illustrates its central design features: sequential Bayesian analyses, dropping or promotion of a trial arm on the basis of these analyses, and introduction of new arms into an ongoing trial. These design features could all be successfully implemented, indicating that at least in the context of simple internet-delivered interventions the leapfrog design is a feasible option for treatment development and optimization.

How can we interpret the results of such a trial and what would be the next steps? We started with an automated, unguided web-based self-help intervention that had never previously been tested in this format, and tested four possible versions of how to implement it; we ended with one ‘winning’ version that was found to be better at reducing anhedonia than an initial control condition, and whose effectiveness was not surpassed by any other version tested. One option would then be to take this winning arm further forwards in the research process, for example testing it in a definitive (superiority or non-inferiority) RCT against established similar low-intensity unguided web-based interventions to see how it compared to these in reducing anhedonia; if pre-planned, such as step could in fact be added seamlessly to the end of the initial leapfrog trial (Blackwell et al., 2019). Alternatively, if we felt that when taking into account all outcome measures (primary and secondary) another arm appeared potentially superior (e.g. CBMv4; see online Supplementary Fig. S2 in the Supplementary Results) we could of course choose this as the arm to take forwards. We could have also decided to continue testing new variants until a certain pre-set threshold in improvement in anhedonia had been achieved (e.g. reflecting a specific level of clinically meaningful improvement; see Table 3), rather than stopping the trial when we did, which was based on the pragmatic rationale of having fulfilled our aim of demonstrating the leapfrog trial features. It is important to note that the current trial was conducted with a lenient set of analysis parameters in order to demonstrate the core leapfrog features; drawing confident conclusions about the efficacy of the interventions tested would require a stricter set of analysis parameters (e.g. higher *N*_min_, *N*_max_, and BF_success_ values, and a lower BF_fail_) with lower associated error rates (see Blackwell et al., 2019, for examples). However, the principles would remain the same, such that the trial would provide a streamlined version of what would normally be a much longer and more drawn-out treatment development process.

**Table 3.** Selected leapfrog trial advantageous design features and potential challenges illustrated in the current trial

Feature	Illustration in trial	Elaboration/Implications
	Leapfrog trial advantageous design features	
Reduced participant numbers	Randomized 35% fewer participants compared to the standard method for treatment development (a series of two-armed trials) with the same power^a^.	Reduced time and participant numbers needed
Rapid dropping of ineffective treatment arms	Treatment arms for which the final effect size (Table 1) showed negligible difference from the relevant control arm dropped soon after reaching Nmin (CBMv1: *n* = 13; CBMv3, *n* = 15)	Fewer participants lost to testing ineffective treatments
Efficient feeding-forward of new data into ongoing trial	Final decisions for the specification of CBMv3 (additional task instructions and rationale) based on relative performance of arms already in the trial (CBMv2 *v.* CBMv1).	Mechanism for real-time data-driven cumulative treatment optimization, eliminating the ‘translational block’ (Blackwell et al., 2019): no need to finish one trial and start another to build on insights from data either coming out of the trial or other latest research findings. However, potential risk of premature inferences from small sample sizes (depending on the chosen BF thresholds).
	Design of CBMv4, introduced partway through the trial, informed by the data accumulated until then (attrition and participant feedback).	
Demonstration of specificity of intervention effects	CBMv2 can be compared to CBMv1 post-hoc and shown superior: BF = 45.42, *d* [95% CIs] = 0.77 [0.30–1.25] (using only those participants who could have been randomized to either arm).	Can demonstrate specificity of intervention (i.e. efficacy is not just non-specific effects) without problematic (Blackwell, Woud, and MacLeod, 2017) placebo/sham intervention conditions
The more effective a treatment is, the more participants will tend to be randomized to that arm.	Trial arms into which the most participants were randomized were those that appeared most effective: CBMv2 and CBMv4.	Particularly advantageous from an ethical perspective, especially if design incorporated into routine care setting.
	Potential challenges and additional factors to consider	
Recruitment *v.* participant number efficiency trade-off	Delay between randomization and providing outcome data: Some participants still completing treatment when BF boundary reached and arm dropped (see Fig. 2; only data from 152 out of 188 participants randomized needed in sequential analyses)	Trade-off between efficiency in terms of speed (fast recruiting) *v.* efficiency of participant numbers (slower or phased recruiting).
		Data from additional participants not ‘wasted’, but informs final BF and effect size calculations (see Table 1). However, the additional data could hypothetically contradict the decision made about dropping or keeping an arm.
Handling of missing data^b^	Used simple ITT approach as in standard RCT to handle missing data	Arms with high attrition have reduced power to hit BF boundaries, increasing risk of large participant numbers ‘wasted’ testing high-attrition treatments.
	Observed differential attrition across arms, higher than predicted levels	Consider more sophisticated approaches, e.g. additional ‘failure’ criterion based on attrition?
Randomization process	Used simple stratified randomization, new randomization sequence naïve to previous allocations generated each time arm composition changed → accumulation of small imbalances over course of trial (CBMv2 only *n* = 36 as CBMv4 hit *N*_max_)	Consider more sophisticated randomization process, e.g. unbalanced randomization at start of new randomization sequence until sample sizes in arms balanced.
Adaptations over time	Kept fixed trial parameters (*N*_min_, *N*_max_, BF_fail_, BF_success_) over whole trial → reduced power to detect improvements once initial control arm replaced by CBMv2.	Consider planning change of trial parameters/primary outcome after control arm replaced increase sensitivity to smaller effect size improvements/avoid ceiling effects.

a 55% power to find *d* = 0.5 at *p* < 0.09 with 25% missing data requires 36 participants per arm. For a series of four 2-arm trials this means 36 × 2 × 4 = 288 participants in total. See online Supplementary Results for full details.

b See Blackwell et al. (2019) for further discussion of missing data.

The current trial illustrates some of the advantageous features of the leapfrog design for treatment development, as well as some additional challenges and factors researchers may wish to consider in optimizing its application in their own research, summarized in Table 3. However, there are limitations to what we can infer about the leapfrog design from the current trial. For example, the design was applied to a simple internet-based intervention, and there will be additional practical issues to consider in other contexts, such as face-to-face therapy delivery (Blackwell et al., 2019). We also used relatively loose inclusion criteria to facilitate recruitment as our main aim was demonstrating the trial design and its implementation, but for full-scale trials it may be preferable to achieve a more precisely-specified sample. Further, the trial was designed and conducted by developers of the leapfrog design, which introduces a potential bias in assessing its success. Finally, as the trial was conducted on small scale over a limited time frame, additional planning considerations may be required if it is used over longer periods of time, for example as a ‘perpetual trial’ (e.g. Hobbs et al., 2018), such as adjustments to the analysis parameters or primary outcome (see Table 3).

In the current study we demonstrated one specific application of the leapfrog design: a treatment development process aiming to find an efficacious version of a new intervention via testing several different variants. However, via application of the basic principles as illustrated here the design could be applied in many ways across the translational spectrum, from pre-clinical work (e.g. developing optimal versions of experimental paradigms or measures) to large clinical trials testing treatment selection algorithms; the principles could also be extended to non-inferiority or equivalence testing and more complex treatment outcomes such as rate of change or cost-effectiveness (see Blackwell et al., 2019, for further discussion). Despite the advantages provided by the design, there are caveats to its use, for example that sequential analyses can lead to biased effect size estimates (Schönbrodt et al., 2017). Further, a researcher needs to be extremely cautious about making simple indirect comparisons between arms that were not in the trial concurrently, as interpretation may be confounded by e.g. seasonal or history effects (see Blackwell et al., 2019, for further discussion; and Marschner and Schou, 2022, for one potential approach to such comparisons). Additionally, although the use of Bayes factors, which are relatively straightforward to compute, makes the design simpler to implement than approaches using more complex analytic approaches such as Bayesian posterior predictive probability (Hobbs et al., 2018) or continuous adjustment of randomization weights (Wason & Trippa, 2014), there is still the need for researchers to familiarize themselves with what might be a new statistical approach before they might feel comfortable to use the design. Similar designs may also be possible using null hypothesis significance testing (i.e. *p* value based) approaches (see Hills and Burnett, 2011, for one example), if ad-hoc adjustments to the *p* value thresholds are incorporated to control for the impact of repeated statistical testing.

The leapfrog design was conceived and developed in the context of a changing landscape for psychological treatment development: For many disorders the most crucial questions are no longer about whether we can develop an efficacious psychological intervention at all, but rather how we can address the need to increase efficacy and accessibility still further, for example via improved targeting of refractory symptoms, tailoring of treatments to individuals, reducing relapse, and innovating in our basic conceptions of psychological interventions and methods of treatment delivery (Blackwell & Heidenreich, 2021; Cuijpers, 2017; Holmes et al., 2018). Addressing these kinds of questions requires moving beyond the standard RCTs that have been the main driver of efficacy research to date, and the last few years have accordingly seen increasing application of more sophisticated trial methodologies (e.g. Collins, Murphy, and Strecher, 2007; Kappelmann, Müller-Myhsok, and Kopf-Beck, 2021; Nelson et al., 2018; Watkins et al., 2016) in psychological treatment research. Within this context, the leapfrog design offers a means to streamline treatment development and optimization, enabling more rapid and resource-efficient progress. The core features of the leapfrog design provide a simple flexible framework that can be built upon to address a wide range of research questions, from simple head-to-head comparisons of treatment variants to more sophisticated questions about sequencing, tailoring, or targeting of interventions (Blackwell et al., 2019). Building on this foundation provides the opportunity to move into a new phase of psychological treatment research, one in which the pace of treatment development can start to match the need for improved outcomes in mental health.

## Footnotes

* The notes appear after the main text.

## Notes

1 The original inclusion criteria additionally specified scoring at least 1 on item 13 (General Interest) of the QIDS, to indicate at least mild levels of anhedonia, but due to a typographic error in the computer code this aspect of the inclusion criteria was not implemented (see online Supplementary Methods for further details).

2 We did not wait for another arm to drop out before introducing CBMv4 (as planned in the trial protocol), but introduced it earlier, as this would make further progress of the trial more efficient in case there was a long delay before another arm hit a BF boundary (see online Supplementary Methods for further elaboration).

3 As the process of ending the trial was not automated, a small number of additional participants were randomized into the trial after CBMv4 hit *N*_max_ (see online Supplementary Methods for further elaboration).

## Supplementary material

For supplementary material accompanying this paper visit https://doi.org/10.1017/S0033291722003294. click here to view supplementary material
