# Supplementary material for: Demonstration of a ‘leapfrog’ randomized controlled trial as a method to accelerate the development and optimization of psychological interventions
Source: Psychol Med. 2022 Nov 4;53(13):6113–23. doi: 10.1017/S0033291722003294 (PMC10520605; doi:10.1017/S0033291722003294)
Supplement: Supplementary file 1 [file S0033291722003294sup.zip › S0033291722003294sup001.docx]

# Supplementary Methods

Contents

[Supplementary Methods 1](#_Toc113972067)

[Amendments to, Deviations from, and Areas Requiring Clarification in the Study Protocol 2](#_Toc113972068)

[Non-Application of the QIDS Anhedonia item Inclusion Criterion 2](#_Toc113972069)

[Earlier Inclusion of Final Training Arm (CBM v4). 2](#_Toc113972070)

[Change to the Specification of CBM v4 3](#_Toc113972071)

[Addition of Instruction about Device Compatibility 3](#_Toc113972072)

[Clarification of Operationalisation of Nmax 3](#_Toc113972073)

[Aggregation of Data for Final Analyses 4](#_Toc113972074)

[Additional Participant Recruitment Information 4](#_Toc113972075)

[Advertising Routes 4](#_Toc113972076)

[Participant Targeting 5](#_Toc113972077)

[Advert Content and Participant Information 6](#_Toc113972078)

[Non-Eligible Participants 7](#_Toc113972079)

[Study Platform 7](#_Toc113972080)

[Measures 8](#_Toc113972081)

[Dimensional Anhedonia Rating Scale (DARS; Rizvi et al., 2015; Wellan, Daniels, & Walter, 2021). 8](#_Toc113972082)

[Quick Inventory of Depressive Symptomatology (QIDS; Roniger, Späth, Schweiger, & Klein, 2015; Rush et al., 2003) 9](#_Toc113972083)

[GAD-7 (Spitzer, Kroenke, Williams, & Lowe, 2006) 10](#_Toc113972084)

[Positive Mental Health Scale (PMH; Lukat, Margraf, Lutz, van der Veld, & Becker, 2016) 10](#_Toc113972085)

[Ambiguous Scenarios Test for Depression (AST; Rohrbacher & Reinecke, 2014) 10](#_Toc113972086)

[Spontaneous Use of Imagery Scale (SUIS; Reisberg, Pearson, & Kosslyn, 2003). 10](#_Toc113972087)

[Prospective Imagery Test (PIT; Stöber, 2000) 11](#_Toc113972088)

[Credibility/ Expectancy Questionnaire (CEQ; Devilly & Borkovec, 2000; Riecke, Holzapfel, Rief, & Glombiewski, 2013) 11](#_Toc113972089)

[Negative Effects Questionnaire (NEQ; Rozental et al., 2019) 11](#_Toc113972090)

[Email Reminders for Questionnaires 12](#_Toc113972091)

[Interventions 12](#_Toc113972092)

[Training Scenarios 12](#_Toc113972093)

[General Training Session Structure 13](#_Toc113972094)

[CBM v1 14](#_Toc113972095)

[CBM v2 15](#_Toc113972096)

[CBM v3 15](#_Toc113972097)

[CBM v4 17](#_Toc113972098)

[Randomization and Allocation Concealment 18](#_Toc113972099)

[Adverse Events: Reliable Change Indices 18](#_Toc113972100)

[References 19](#_Toc113972101)

## Amendments to, Deviations from, and Areas Requiring Clarification in the Study Protocol

Non-Application of the QIDS Anhedonia item Inclusion Criterion

In the original protocol we planned to use an inclusion criterion of scoring at least 1 on the anhedonia item (item 13) of the Quick Inventory of Depressive Symptomatology (QIDS). However, during the review process for the paper we realised that this inclusion criterion had not been applied: The process of judging participants to be eligible or otherwise was automated within the trial platform and a typographic error in the line of code intended to apply this inclusion criterion meant that it was not checked or implemented. Hence participants scoring 0 on this item (*n* = 34) were included in the trial if they met the other inclusion criteria. As the main purpose of this criterion was to reduce the chance of ceiling effects for our primary outcome measure, and thus facilitate our aim of demonstrating the leapfrog trial design, the main effect will have been to make it more difficult for trial arms to demonstrate superiority and therefore it does not affect the conclusions drawn about the leapfrog trial design itself.

Earlier Inclusion of Final Training Arm (CBM v4).

In the original protocol we stated that to reduce the number of participants required, a maximum of 3 arms (including the comparison arm) would be included at any one time, and an additional arm would only be included as a replacement for an arm dropping out. After dropping of CBM v1 due to hitting BF_fail_, it was decided to introduce the final training arm (CBMv4) earlier, before another arm had dropped out (i.e. so that there would be four parallel arms) as this would be more efficient in terms of numbers of participants required for the trial in case it took a long time for another arm to drop out.

Change to the Specification of CBM v4

Originally the last new training arm to be introduced (CBM v4) was planned to test out repetition of training scenarios (see study protocol, section 6.2.2.2). However, data coming from CBM v1 and CBM v2 (i.e., rate of drop-out and participant feedback) suggested that many participants found the training schedules in the first two weeks too intensive and the training sessions too long. Therefore it seemed that it was more important to increase adherence to and acceptability of the training schedule than to make relatively subtle changes to the internal content of the training sessions as originally planned (e.g. repetition of scenarios). It was therefore decided that the last arm to be introduced (CBM v4) would be designed to try to have a more acceptable training schedule (see the detailed intervention sections below for details).

Addition of Instruction about Device Compatibility

At the time that CBM v2 hit BF_success_, the information about the study (on the information page where participants registered, and at the start of the baseline questionnaires) was updated to inform participants that the training could not be completed on an iPad or iPhone, following feedback from a participant. The problem appeared to be related to iPads and iPhones blocking autoplay or preloading of training scenarios, but was judged to be not resolvable while the study was ongoing due to lack of availability of an iPad for repeated testing and trouble-shooting.

Clarification of Operationalisation of Nmax

As CBM v4 hit *N*_max_ (*n* = 40), CBM v2 (the current control arm) included only 34 participants, due to small imbalances in the randomization that had accrued over the course of the trial. It became apparent at this time that the simulations used to plan the trial had assumed equal sample sizes, and hence *n* = 40 in both arms at *N*_max_. Hence, stopping with only 34 participants in the control arm would result in less power than that assumed by the simulations. Following discussion amongst the research team we decided to follow our specification of *N*_max_ as specified in the trial protocol, i.e. the treatment arm reaching *N*_max_ without any reference to the sample size achieved in the control arm. However, in future trials it would be preferable to plan for potential imbalanced randomization, for example specifying *N*_max_ in a similar way to *N*_min_, that is, a sample size that needs to be reached in both arms, or specifying it as the total sample size summed across both arms (e.g. in our case, this would be *N* = 80). In practice a few additional participants were randomized into both arms after CBM v4 reached *N*_max_, due to a delay in recognising and making this decision, and because after the study platform was updated so that no new participants could register for the trial, there were a small number of participants who had registered but not yet completed baseline measures and been randomized. These already-registered participants were allowed to continue with the trial, and hence were randomized after recruitment had ended.

Aggregation of Data for Final Analyses

The original protocol stated that raw datafiles would be downloaded from the RUB server as .csv files for the final analyses, but in practice to streamline the data aggregation process the data was pulled directly off the server into RStudio using the package httr (Wickham, 2017).

## Additional Participant Recruitment Information

### Advertising Routes

The study was advertised online via: members of the study team posting on social media (Facebook, Twitter, Instagram, Whatsapp groups); physical fliers displayed or handed out e.g. in local shops, on the university campus; a press release via Ruhr-Universität Bochum; a brief radio interview with two of the study researchers (SEB, MLW) on a local radio station (Radio Bochum), which then linked to the press release; posts on the website of the Mental Health Research and Treatment Center and other websites with study listings or discussion forums; paid adverts on Google and Facebook. Recruitment was tracked via url parameters at the end of the links used to direct people to the study. According to these url parameters, the number of participants who came from the various sources was as follows [in the format, total number registered (number randomized)] : Department website: 5 (5); Facebook posts: 6 (4); Instagram: 15 (8); Twitter: 14 (11); Whatsapp groups: 5 (4); Facebook paid adverts: 72 (49); Google adverts: 9 (8); Fliers: 1 (1); Press release: 128 (77); Unknown: 87 (20). However, several sources (e.g. the radio advert, some posts in forums and on social media) linked to the press release and so numbers for the press release will include some indirect referrals from other sources. Further, it may be that links originally shared on one forum (e.g. Facebook) were then passed on to other participants via other routes (e.g. via a friend).

Participant Targeting

Targeting of advertisements to potential participants who might be experiencing symptoms of depression and interested in taking part was implemented in a number of ways. For the Google adverts, keywords such as ‘depression’, ‘online therapy’, ‘depression self-help’ and ‘help with depression’ were selected, such that the advert might be shown to people searching for these and similar terms in Google. The Google adverts were also only shown to people with an IP address in Germany. For paid Facebook adverts, adverts were shown only to people aged 18 or over, living in Germany or Austria, and with their language set to German. However, no further detailed targeting was used as selecting potentially depression-relevant interests (e.g. depression self-help groups) could lead to excluding too many potential participants. Targeted non-paid advertising on Facebook was achieved via posting information about the study in German-language mental health-related forums or groups, where such posting was allowed. Other targeted online advertising for the study was via posting on German-language mental-health related websites/forums where this was allowed. Otherwise, maximising the chance that people who clicked on the adverts would be interested in the study was achieved via the advert content itself.

Advert Content and Participant Information

The study was advertised as “Mentales Imaginationstraining Online” [Mental imagery training online], or “MIO-Studie” for short, and adverts used phrases such as “Do you feel low and depressed? Take part in our research study and test a new online training program to help yourself and others!”. The participant information sheet explained that the study was testing a ‘brain-training’ program that we thought could potentially reduce symptoms of depression and anxiety, but which we wished to improve. Participants were informed that the study involved completing weekly questionnaires over four weeks, and that in addition they may be asked to complete one of several potential versions of the training. The training and the potential kinds of differences between the different versions (e.g. number of sessions, session length) were explained in general terms, but precise details of the different training arms not elaborated upon. Participants were informed that the study was not suitable for people experiencing high levels of distress and needing urgent help, and that although we hoped the training would be useful, it was not equivalent to psychological therapy and not suitable for people who felt they were in need of psychological therapy. Information about how to access psychological therapy or other help was provided. The full information sheet and consent form can be found in the appendix to the protocol available on the open science framework (<https://osf.io/8mxda/>).

Non-Eligible Participants

Participants who registered for the study but who on completing the QIDS at baseline did not meet the inclusion criteria were not given any feedback about this, but rather were able to continue and complete all study procedures (to reduce the chance that they might re-register but provide an artificially inflated QIDS score in order to take part). However, these participants were randomized to a trial arm via a simple randomization procedure independently of eligible participants and their data was not included in analyses.

## Study Platform

The online study platform, which was hosted on a secure server based at the RUB Mental Health Research and Treatment Center, was programmed by SEB using JavaServerPages, with a Java servlet running on Apache Tomcat and connected to a MySQL database, and with study procedures implemented client-side using HTML, CSS, and Javascript. Participants accessed the study from their internet browser via https. Detailed information about the software and its implementation, as well as the source code, can be found on study’s webpage on the open science framework (<https://osf.io/8mxda/>).

The website for the study provided a platform for participants to complete questionnaires and training sessions. On logging in, participants were presented with a ‘home’ page with information about when the next questionnaires or training sessions were due, and a button to complete either questionnaires or training if these were currently available.

A ‘My Progress’ page provided participants with information about their progress through the study. One panel showed the schedule of training sessions and questionnaires completed or due for completion, laid out as a four-week calendar. A second panel showed the same schedule but laid out as a simple text-based list. A third panel showed the participant a graphical display their scores on the weekly questionnaires (QIDS, GAD-7, PMH), including some explanatory information about how these can be interpreted (e.g. categories of depression severity). For participants in a training condition, a graphical display of the participant’s vividness scores for each session (minimum, maximum, and mean per session) was also provided.

An ‘Information’ page provided participants with further information and help possibilities. One panel displayed the participant information sheet with the option to download it as a pdf. A second panel provided participants with options to change their password or times at which they received email reminders about training sessions and questionnaires. A third panel provided ‘Frequently Asked Questions’, such as what a participant should do if they missed a training session, or if they had trouble imagining the training scenarios, as well as more general information about seeking help in times of crisis. A fourth panel provided a secure messaging system. Participants could write messages to the research team, which were stored in encrypted form on the study server. Researchers could read the messages and reply via logging into the study website via a researcher interface. Thus participants could ask questions and receive responses anonymously. The participant/ researcher received a notification email if they had received a message, but to read the message itself they had to log into the study website.

## Measures

### ***Dimensional Anhedonia Rating Scale*** (DARS; Rizvi et al., 2015; Wellan, Daniels, & Walter, 2021)***.***

The DARS is a 17-item self-report questionnaire in which participants were asked to type in 2 or 3 examples of favourite activities across 4 domains (hobbies/leisure, social, food/drink, sensory experiences) and then rate these on 5-point scales assessing interest/desire, anticipated consummatory pleasure, motivation, and effort. Higher scores indicate lower levels of anhedonia. At post-intervention participants were shown the activities they provided at baseline and asked to rate these again. Internal consistency (Cronbach’s alpha) in our sample was excellent at both baseline, *α* = 0.92, 95% CIs [0.90,0.93], and post-intervention, *α* = 0.94 [0.93,0.95].

### ***Quick Inventory of Depressive Symptomatology*** (QIDS; Roniger, Späth, Schweiger, & Klein, 2015; Rush et al., 2003)

The QIDS is a brief self-report measure of depressive symptoms that was designed to assess core criterion DSM-IV symptoms of depression, asking participants about their experience of each symptom over the past 7 days. We used a 15-item version in which the suicide item was omitted, as in the anonymous online format of the trial risk assessment and management was not feasible. The German translation provided by the scale publishers (http://www.ids-qids.org/) was used. Internal consistency in our study (Cronbach’s alpha [95% CIs]) was as follows: Baseline: 0.66 [0.58,0.72]; Post week 1: 0.71 [0.65,0.77]; Post week 2: 0.75 [0.70,0.80]; Post week 3: 0.80 [0.76,0.84]; Post-intervention: 0.79 [0.75,0.83].

When aggregating data for the final analyses, it was discovered that the process for scoring the QIDS automatically within the online study platform (used for stratification in the randomization process, judging eligibility, and providing feedback to participants) contained an error, in that the score from items 6 (reduced appetite) / 7 (increased appetite) and items 8 (weight gain) / 9 (weight loss) were both used, rather than just the maximum score of these items. This meant that two participants (one in monitoring, one in CBM v2) would not have met the QIDS ≥ 6 inclusion criterion had the correct scoring been in place, and 3 participants were placed in the high depression (QIDS > 9) stratum who would have been placed in the low depression stratum had the scoring process been correct. The QIDS scoring was corrected for the purposes of the analyses presented in this paper.

### ***GAD-7*** (Spitzer, Kroenke, Williams, & Lowe, 2006)

The GAD-7 is a brief 7-item measure of generalized anxiety symptoms, in which participants are asked to rate the frequency of each symptom over the past two weeks. The German translation by Lowe et al. (2008) was used. Internal consistency in our study (Cronbach’s alpha [95% CIs]) was as follows: Baseline: 0.84 [0.80,0.87]; Post week 1: 0.84 [0.81,0.87]; Post week 2: 0.81 [0.77,0.85]; Post week 3: 0.85 [0.82,0.88]; Post-intervention: 0.87 [0.85,0.90].

### ***Positive Mental Health Scale*** (PMH; Lukat, Margraf, Lutz, van der Veld, & Becker, 2016)

The PMH (original in German) is a 9-item questionnaire assessing various aspects of positive mental health such as experience of positive emotions and enjoyment of one’s life. Internal consistency in our study (Cronbach’s alpha [95% CIs]) was as follows: Baseline: 0.90 [0.88,0.92]; Post week 1: 0.92 [0.90,0.93]; Post week 2: 0.92 [0.91,0.94]; Post week 3: 0.93 [0.92,0.94]; Post-intervention: 0.94 [0.93,0.95].

### ***Ambiguous Scenarios Test for Depression*** (AST; Rohrbacher & Reinecke, 2014)

The AST was included as a measure of negative interpretation bias (one of the mechanisms theoretically targeted by the imagery CBM intervention). Participants read 15 ambiguous scenario descriptions and were asked to imagine each scenario happening to them personally. They then rated how pleasant/unpleasant the imagined scenario was on a scale from -5 (very unpleasant) to +5 (very pleasant). Two parallel forms of the AST (‘A’ and ‘B’ forms) were used, with simple counterbalancing (alternating between odd and even participant numbers) used to allocate participants to complete either A at pre-training and B at post-intervention or vice versa. Internal consistency in our study (Cronbach’s alpha [95% CIs]) was as follows: Version A: Baseline: 0.81 [0.75,0.86]; Post-intervention: 0.90 [0.87,0.93]; Version B: Baseline: 0.80 [0.73,0.85]; Post-intervention: 0.89 [0.86,0.92].

### **Spontaneous Use of Imagery Scale** (SUIS; Reisberg, Pearson, & Kosslyn, 2003)**.**

The SUIS is a measure of the tendency to experience (non-emotional) imagery in everyday life. The (12-item) German translation by Görgen et al. (2016) was used. Internal consistency in our study (Cronbach’s alpha [95% CIs]) was 0.75 [0.69,0.80].

### **Prospective Imagery Test** (PIT; Stöber, 2000)

The PIT is a measure of the vividness with which people can imagine positive or negative events happening in their future (a putative mechanism targeted via imagery CBM). Participants read a list of hypothetical positive and negative future scenarios and were asked to form a mental image of each, before rating how vivid their image was from 1 (*not at all vivid*) to 5 (*very vivid*). The German version by Morina et al. (2011) was used. Two 10-item parallel forms of the PIT (‘A’ and ‘B’ forms) were used, with simple counterbalancing (alternating between odd and even participant numbers) used to allocate participants to complete either A at pre-training and B at post-intervention or vice versa. Internal consistency in our study (Cronbach’s alpha [95% CIs]) was as follows: Positive items, version A: Baseline: 0.82 [0.76,0.86]; Post-intervention: 0.85 [0.80,0.89]; Positive items version B: Baseline: 0.67 [0.56,0.76]; Post-intervention: 0.86 [0.82,0.90]; Negative items, version A: Baseline: 0.72 [0.64,0.80]; Post-intervention: 0.66 [0.55,0.75]; Negative items version B: Baseline: 0.64 [0.53,0.74]; Post-intervention: 0.75 [0.68,0.81].

### ***Credibility/ Expectancy Questionnaire*** (CEQ; Devilly & Borkovec, 2000; Riecke, Holzapfel, Rief, & Glombiewski, 2013)

The CEQ was scored as per the original instructions (Devilly & Borkovec, 2000). That is, participants’ score on each item was converted to a z-score based on the whole (randomized) sample, and then the three credibility items and the three expectancy items summed to form the credibility and expectancy scores, respectively.

### **Negative Effects Questionnaire** (NEQ; Rozental et al., 2019)

The NEQ is designed to measure potential unwanted and adverse effects of psychological treatments. The short (20-item) German version provided via the scale authors was used, with terms ‘therapy’ and ‘therapist’ changed to more appropriate terms for this study such as ‘study’ and ‘researchers’.

### **Email Reminders for Questionnaires**

For the questionnaires due at the end of the first, second, and third weeks, participants received up to two reminder emails on the day the questionnaire was due and up to two reminder emails the following day. For the final set of questionnaires, participants received up two emails the day the questionnaires were due, and the following day, and then one email three (am), four (pm), and six (am) days after the emails were due. Once participants had completed the questionnaires they received no further reminder emails. The default times for the reminder emails were 6am and 6pm, but participants could change these if they wished.

## Interventions

This section describes the interventions in more detail. All interventions took place over a four-week period as described in the main paper.

### Training Scenarios

As in previous studies (e.g., Blackwell et al., 2015; Westermann et al., 2021), each training scenario consisted of a brief (e.g. 10 – 15 second) description of an everyday situation, structured so that started ambiguous as to how they might end, but then always ending positively, for example “You have just woken up and lie in bed thinking about the upcoming day. As you consider what the day might bring, you feel more and more *excited*” (positive resolution in italics). A total of 524 training scenarios were recorded (plus 13 practice scenarios), of which 356 were taken from a previous study (Westermann et al., 2021), and 168 were newly generated for the current study. All scenarios were newly recorded for the current study, divided equally between four voices (2 male, 2 female). Scenarios were randomly allocated to each training session prior to the start of the study, such that all participants heard the same training scenarios in each session but in a (different) random order within the session. No scenarios were repeated during the training, although if participants quit a session and resumed it later on the order of scenarios for that session was randomized afresh, such that it was possible for participants to hear a scenario more than once if they did not complete a training session in one attempt.

Presentation of each training scenario started with a screen saying “Close your eyes. Imagine.” for 1500 ms, followed by a black screen during which the scenario was played. 2000 ms after the scenario had finished playing, a beep sounded and participants were asked to rate how vividly they had imagined the scenario on a scale from 1 (*not at all vividly*) to 7 (*extremely vividly*). After the participant had made their rating by clicking a corresponding button, the program moved directly on to the next scenario (or the break / end of session screens if applicable).

### General Training Session Structure

In general, each training session started with an introductory overview page followed by ratings of state mood, using the 5 positive items and 5 negative items from the short Positive and Negative Affect Schedules (PANAS; Mackinnon et al., 1999). Participants were then shown instructions for the training, followed by a practice scenario, which was the same in structure to other training scenarios. After the practice scenario, participants were presented with some descriptive information about the kinds of details they might have imagined (e.g. sights, sounds, feelings), and were invited to try to imagine the practice scenario again incorporating all these elements. Each session used a different practice scenario.

Participants then completed the sets of training scenarios. In between each set of scenarios, participants were shown a tabular display of their minimum, maximum, and average vividness ratings for all sets completed so far that session, and encouraged to reflect on the scores, what had helped or hindered them in generating vivid imagery, and how they could try to improve on this in the subsequent set (adapted from Westermann et al., 2021).

At the end of the sets of scenarios, participants repeated the PANAS mood ratings and saw a final table of their vividness ratings over the whole session, after which the session was completed. Participants could exit a session at any time, and provided that they had completed at least one training scenario they could resume it where they had left off at a later time point (while the session was still available to complete).

### CBM v1

CBM v1 was designed as a ‘standard’ version of imagery CBM, close to that implemented in previous studies (albeit adapted for automated unguided anonymous web-based delivery). It comprised an introductory session followed by two weeks of 4 sessions of 40 scenarios (15-20 mins), as in Westermann et al. (2021), and then two weeks of two 2 sessions each, as in the last two weeks of the 4-week schedule used by Blackwell et al. (Blackwell et al., 2015; albeit with 40 rather than 64 training scenarios per session). Instructions and other aspects of the training were be adapted from that used by Westermann et al. (2021). The introductory session included an extended introduction to mental imagery, with several practice examples, followed by 4 blocks of 5 scenarios.

Participants received up to two reminder emails on the day of each training session, a first at 6am, and if the session was not yet completed, another at 6pm. If they did not complete the session, they would receive a reminder email at 6am the next morning, unless there was another session already scheduled for that day. Participants were able to change the time of these reminder emails. Sessions were scheduled to become available on fixed days, but remained available to complete until the end of the training week that they were scheduled in. Participants had to complete the introductory session in order to be able to proceed with other training sessions, but otherwise were not able to complete earlier-scheduled sessions in order to complete later sessions. All sessions after the introductory one were identical in their general structure, with the only difference being the practice and training scenarios used.

### CBM v2

CBM v2 represented a first attempt to improve on the ‘standard’ imagery CBM, via incorporating features based on feedback from participants in previous studies (e.g., Blackwell et al., 2015; Blackwell & Holmes, 2010). It used an identical schedule of sessions as CBM v1, but additionally included a more extended ‘treatment rationale’ for the training (typically this is not included in standard imagery CBM, but would seem a necessary adaptation in order for successful delivery as an unguided intervention). Further, at the end of each training sessions participants were presented with instructions and suggestions to practice recalling and rehearsing the training scenarios in daily life (e.g. when they encounter similar situations). This was presented as ‘tips’ to ‘make the most of the training’ that were derived from previous studies. In order to reinforce these instructions, at the start of each session participants provided ratings of how frequently they had thought about the training in daily life, both deliberately and involuntarily. These adaptations were intended to enhance transfer of training (Blackwell, 2020), and based on anecdotal reports from previous studies, in which some participants reporting beneficial effects of imagery CBM described using recall and rehearsal of training scenarios in daily life as spontaneously adopted strategy to improve their mood (Blackwell & Holmes, 2010, 2017).

### CBM v3

CBM v3 attempted to improve upon the standard imagery CBM by using a different kind of training schedule, specifically more frequent (twice per day as per Browning, Holmes, Charles, Cowen, & Harmer, 2012) but shorter training sessions. It was thought that this might be both more convenient and lead to better learning (spaced rather than massed learning via distributing the training scenarios into a greater number of sessions). The introductory training session included 4 sets of 5 scenarios, as for CBM v1 and CBM v2. However, thereafter, each of the four training weeks comprised 5 days on which training sessions were scheduled followed by two days on which no training was scheduled. Two sessions were scheduled on each training day, each comprising two blocks of six scenarios (~ 5 mins).

It was planned that the specifications of CBM v3 would be informed by earlier results in the trial, specifically that it would build on whichever of CBM v2 or CBM v1 had a larger Bayes factor vs. Monitoring when CBM v3 was introduced. As CBM v3 was introduced when CBM v1 hit BF_fail_, it built on CBM v2, using its features such as the extended rationale, instructions to think about scenarios in between sessions, and asking participants to rate how much they had thought about the training scenarios in between sessions (at the start of one of the two sessions scheduled on a particular day).

CBM v3 used a different pattern of email reminders about training sessions compared to CBM v1 and CBM v2: On the first training day of each week, participants received an email announcing the start of the training week and encouraging them to complete as many of the training sessions as they could, as well as to plan this and set themselves reminders. Participants then did not receive any further reminder emails until the morning of the last training day of the week, announcing that they had come to the end of the training week and suggesting that they could use the following days to catch up on training if necessary. The idea here was to give participants more autonomy and responsibility about how they organised the training, and to prevent them feeling overwhelmed by reminder messages. The emails also explicitly told participants that they should just do as much or as little training as they were able to, and regardless of how much they managed we would value them completing the final set of questionnaires for the study.

### CBM v4

CBM v4 was specifically designed to try to increase engagement and reduce attrition, following the observation that CBM v1 and CBM v2 had higher attrition rates than planned for, and that some participants were providing feedback that there were too many sessions and they were too long. Participants appeared to drop out relatively early in the study, suggesting that they might find the intensive training schedule of the first week too overwhelming. As with CBM v3, CBM v4 was based on CBM v2 in terms of task rationale and instructions to recall and rehearse training scenarios in between sessions. In addition, instead of one introductory session followed by 12 sessions over 4 weeks (4 per week in the 1st two weeks, 2 per week in the last two weeks), there was one introductory session, and then 11 sessions over 4 weeks (2 in the first week, 3 in subsequent weeks), for a more gradual start into the training schedule. The length of the sessions was shortened from 40 scenarios to 32 scenarios per session (4 sets 8 of scenarios), and the introductory session included 10 scenarios (2 sets of 5) instead of 20. The task instructions at the start and end of the training sessions were also made more varied, for example, not always repeating the same brief introduction to each session, but alternating between presenting it in full, in part, or not at all. Further, a “Training tip of the day” was added to the start of each session. This was different for every session and repeated a key instruction for the training or about the study (e.g. that it is very helpful for the research to complete the questionnaires even if participants do not want to complete training sessions). In some sessions the tip of the day, or instructions at the end of the session, emphasised that it may take time for participants to feel benefits and encouraged them to continue with the training (and the study more broadly) even if they felt it was not benefiting them (i.e. to manage participants‘ expectancy about how quickly they might expect to feel benefits).

CBM v4 used the same pattern of email reminders about training sessions as CBM v1 and CBM v2, but the reminder emails also included a sentence reminding participants that if they did not have time to complete a whole session they could just log in and do as much of the session as they had time for.

## Randomization and Allocation Concealment

Random sequences were generated using the java.util.concurrent.ThreadLocalRandom class within the online study platform (programmed by SEB). Whenever the composition of arms changed (e.g. arm added, arm removed), any unused allocations from the current sequences were deleted, and a new set of randomization sequences was generated so that there was always an equal probability of being randomized to any one trial arm. For the purpose of allocation concealment, participants were randomized after completing baseline measures and did not know about prior allocations. Thus there was no way that participants could predict their allocation, or that baseline measure completion could be biased by knowledge of allocation. Participant recruitment, baseline measure completion, and allocation occurred remotely, and any researchers in contact with participants at this stage would not have access to randomization information, ensuring adequate allocation concealment on the part of relevant researchers.

## Adverse Events: Reliable Change Indices

Reliable Change Indices (RCIs) were calculated as follows: GAD-7: RCI = 4.17, based on SD = 3.88 and Cronbach’s alpha = 0.85 (Hinz et al., 2017); QIDS: RCI = 6.49, based on SD = 4.88 and Cronbach’s alpha = 0.77 (Roniger et al., 2015).

# References

Blackwell, S. E. (2020). Clinical efficacy of cognitive bias modification interventions. *The Lancet. Psychiatry*, *7*(6), 465–467. https://doi.org/10.1016/S2215-0366(20)30170-X

Blackwell, S. E., Browning, M., Mathews, A., Pictet, A., Welch, J., Davies, J., … Holmes, E. A. (2015). Positive imagery-based cognitive bias modification as a web-based treatment tool for depressed adults: A randomized controlled trial. *Clinical Psychological Science*, *3*(1), 91–111. https://doi.org/10.1177/2167702614560746

Blackwell, S. E., & Holmes, E. A. (2010). Modifying interpretation and imagination in clinical depression: A single case series using cognitive bias modification. *Applied Cognitive Psychology*, *24*(3), 338–350. https://doi.org/10.1002/acp.1680

Blackwell, S. E., & Holmes, E. A. (2017). Brightening the Day With Flashes of Positive Mental Imagery: A Case Study of an Individual With Depression. *Journal of Clinical Psychology*, *73*(5). https://doi.org/10.1002/jclp.22455

Browning, M., Holmes, E. A., Charles, M., Cowen, P. J., & Harmer, C. J. (2012). Using attentional bias modification as a cognitive vaccine against depression. *Biological Psychiatry*, *72*(7), 572–579. https://doi.org/10.1016/j.biopsych.2012.04.014

Devilly, G. J., & Borkovec, T. D. (2000). Psychometric properties of the credibility/expectancy questionnaire. *Journal of Behavior Therapy and Experimental Psychiatry*, *31*, 73–86. https://doi.org/10.1016/S0005-7916(00)00012-4

Görgen, S. M., Hiller, W., & Witthöft, M. (2016). Die Spontaneous Use of Imagery Scale (SUIS)–Entwicklung und teststatistische Prüfung einer deutschen Adaption | Diagnostica | Vol 62, No 1. *Diagostica*, *62*, 31–43. https://doi.org/10.1026/0012-1924/a000135

Hinz, A., Klein, A. M., Brähler, E., Glaesmer, H., Luck, T., Riedel-Heller, S. G., … Hilbert, A. (2017). Psychometric evaluation of the Generalized Anxiety Disorder Screener GAD-7, based on a large German general population sample. *Journal of Affective Disorders*, *210*, 338–344. https://doi.org/10.1016/J.JAD.2016.12.012

Lukat, J., Margraf, J., Lutz, R., van der Veld, W. M., & Becker, E. S. (2016). Psychometric properties of the Positive Mental Health Scale (PMH-scale). *BMC Psychology*, *4*, 8. https://doi.org/10.1186/s40359-016-0111-x

Mackinnon, A., Jorm, A. F., Christensen, H., Korten, A. E., Jacomb, P. A., & Rodgers, B. (1999). A short form of the Positive and Negative Affect Schedule: Evaluation of factorial validity and invariance across demographic variables in a community sample. *Personality and Individual Differences*, *27*(3), 405–416.

Morina, N., Deeprose, C., Pusowski, C., Schmid, M., & Holmes, E. A. (2011). Prospective mental imagery in patients with major depressive disorder or anxiety disorders. *Journal of Anxiety Disorders*, *25*(8), 1032–1037. https://doi.org/10.1016/j.janxdis.2011.06.012

Reisberg, D., Pearson, D. G., & Kosslyn, S. M. (2003). Intuitions and introspections about imagery: The role of imagery experience in shaping an investigator’s theoretical views. *Applied Cognitive Psychology*, *17*(2), 147–160. https://doi.org/10.1002/acp.858

Riecke, J., Holzapfel, S., Rief, W., & Glombiewski, J. A. (2013). Evaluation and implementation of graded in vivo exposure for chronic low back pain in a German outpatient setting: A study protocol of a randomized controlled trial. *Trials*, *14*(1), 203. https://doi.org/10.1186/1745-6215-14-203

Rizvi, S. J., Quilty, L. C., Sproule, B. A., Cyriac, A., Bagby, M. R., & Kennedy, S. H. (2015). Development and validation of the Dimensional Anhedonia Rating Scale (DARS) in a community sample and individuals with major depression. *Psychiatry Research*, *229*(1–2), 109–119. https://doi.org/10.1016/j.psychres.2015.07.062

Rohrbacher, H., & Reinecke, A. (2014). Measuring change in depression-related interpretation bias: Development and validation of a parallel ambiguous scenarios test. *Cognitive Behaviour Therapy*, *43*(3), 239–250. https://doi.org/10.1080/16506073.2014.919605

Roniger, A., Späth, C., Schweiger, U., & Klein, J. (2015). A Psychometric Evaluation of the German Version of the Quick Inventory of Depressive Symptomatology (QIDS-SR16) in Outpatients with Depression. *Fortschritte Der Neurologie Psychiatrie*, *83*(12), e17–e22. https://doi.org/10.1055/s-0041-110203

Rozental, A., Kottorp, A., Forsström, D., Månsson, K., Boettcher, J., Andersson, G., … Carlbring, P. (2019). The Negative Effects Questionnaire: Psychometric properties of an instrument for assessing negative effects in psychological treatments. *Behavioural and Cognitive Psychotherapy*, *47*(5), 559–572. https://doi.org/10.1017/S1352465819000018

Rush, J. A., Trivedi, M. H., Ibrahim, H. M., Carmody, T. J., Arnow, B., Klein, D. N., … Keller, M. B. (2003). The 16-item quick inventory of depressive symptomatology (QIDS), clinician rating (QIDS-C), and self-report (QIDS-SR): A psychometric evaluation in patients with chronic major depression. *Biological Psychiatry*, *54*(5), 573–583. https://doi.org/10.1016/S0006-3223(02)01866-8

Spitzer, R. L., Kroenke, K., Williams, J. B. W., & Lowe, B. (2006). A brief measure for assessing generalized anxiety disorder: The GAD-7. *Archives of Internal Medicine*, *166*, 1092–1097.

Stöber, J. (2000). Prospective cognitions in anxiety and depression: Replication and methodological extension. *Cognition & Emotion*, *14*(5), 725–729. https://doi.org/10.1080/02699930050117693

Wellan, S. A., Daniels, A., & Walter, H. (2021, March 12). *State anhedonia in young healthy adults: Psychometric properties of the German Dimensional Anhedonia Rating Scale (DARS) and effects of the COVID-19 pandemic*. PsyArXiv. https://doi.org/10.31234/osf.io/rvtjm

Westermann, K., Woud, M. L., Cwik, J. C., Graz, C., Nyhuis, P. W., Margraf, J., & Blackwell, S. E. (2021). Feasibility of computerised positive mental imagery training as a treatment adjunct in in-patient mental health settings: Randomised controlled trial. *BJPsych Open*, *7*(6). https://doi.org/10.1192/bjo.2021.1042

Wickham, H. (2017). *httr: Tools for Working with URLs and HTTP. R package version 1.4.1.* Retrieved from https://CRAN.R-project.org/package=httr
