# Supplementary material for: Demonstration of a ‘leapfrog’ randomized controlled trial as a method to accelerate the development and optimization of psychological interventions
Source: Psychol Med. 2022 Nov 4;53(13):6113–23. doi: 10.1017/S0033291722003294 (PMC10520605; doi:10.1017/S0033291722003294)
Supplement: Supplementary file 1 [file S0033291722003294sup.zip › S0033291722003294sup002.docx]

# Supplementary Results

Contents

[Supplementary Results 1](#_Toc113976867)

[Participant Characteristics 2](#_Toc113976868)

[Adherence Data 2](#_Toc113976869)

[Outcome Measures 2](#_Toc113976870)

[Participant Feedback 3](#_Toc113976871)

[Adverse Events and Negative Effects Questionnaire 3](#_Toc113976872)

[Sample Size Comparisons 4](#_Toc113976873)

[References 5](#_Toc113976874)

[Supplementary Table 1. Detailed Participant Characteristics at Baseline. 6](#_Toc113976875)

[Table S2. Detailed adherence data in each trial arm, N (%) unless otherwise indicated. 11](#_Toc113976876)

[Figure S1. Observed means and 95% Confidence Intervals for the outcome variables across all trial arms. 12](#_Toc113976877)

[Table S3. Outcome data: Observed means and standard deviations, as well as number of participants providing outcome data at each timepoint. 13](#_Toc113976878)

[Figure S2a. Observed means and 95% Confidence Intervals for CBM v1 (n = 26) vs. Monitoring (n = 27). 15](#_Toc113976879)

[Figure S2b. Observed means and 95% Confidence Intervals for CBM v2 (n = 29) vs. Monitoring (n = 31). 16](#_Toc113976880)

[Figure S2c. Observed means and 95% Confidence Intervals for CBM v3 (n = 22) vs. CBM v2 (n = 18). 17](#_Toc113976881)

[Figure S2d. Observed means and 95% Confidence Intervals for CBM v4 (n = 43) vs. CBM v2 (n = 36). 18](#_Toc113976882)

[Table S4. Participants responses on the Feedback Questionnaire 19](#_Toc113976883)

[Table S5a. Participant responses on the Negative Effects Questionnaire (all negative effects). 20](#_Toc113976884)

[Table S5b. Participant responses on the Negative Effects Questionnaire including only those negative effects ascribed to the study 23](#_Toc113976885)

## Participant Characteristics

Table S1 provides more detailed demographic and background information about participants in each trial arm.

## Adherence Data

Table S2 provides more detailed adherence data for each training arm over the course of the trial. Although the rate of attrition in the training arms was higher than planned for in the simulations that informed the trial parameters (which went up to 25% attrition), and higher than that for previous imagery CBM studies (most of which had very low attrition rates, e.g., Blackwell et al., 2015; Pictet, Jermann, & Ceschi, 2016), it was not unusually high for an unguided web-based intervention in the context of depression (e.g., Christensen, Griffiths, & Farrer, 2009; Titov et al., 2013)

## Outcome Measures

Figure S1 and Table S3 provide descriptive statistics for the DARS and all secondary outcome measures across the timepoints of the trial. Note that caution must be taken in interpreting outcomes between trial arms, because not all data was collected concurrently. Comparing complete outcome data across arms therefore i) violates the principle of randomization, for example participants in CBM v4 could not have been randomized into monitoring, and ii) introduces differential time of year / history effects. For example, over the initial timecourse of data collection for monitoring, CBM v1, and CBM v2, the weather in Germany changed from cold and rainy to warm and sunny, as the seasons progressed from Spring to Summer. Further, COVID restrictions were relaxed considerably over this time. Conversely, the final timecourse of data collection for CBM v2 and CBM v4 was approaching the end of 2021, associated with the onset of winter and tightening of COVID restrictions. This is perhaps reflected in the difference in magnitude of improvement seen in CBM v2 in Figure S2a and S2b (early in the trial) vs. Figure S2c and S2d (late in the trial).

For the purpose of comparison on all outcome measures, Figure S2(a – d) therefore shows data (observed means and 95% Confidence Intervals) for each individual training conditions vs. their relevant control condition, including only those participants concurrently randomized.

## Participant Feedback

Table S4 shows participant feedback across the different trial arms. The number of participants in each arm who re-registered for further training at the end of their participation were as follows: Monitoring: n = 13 (41.94%); CBM v1: n = 4 (15.38%); CBM v2: n = 11 (16.92%); CBM v3: n = 7 (31.82%); CBM v4: n = 13 (30.23%).

## Adverse Events and Negative Effects Questionnaire

Two participants showed reliable deterioration on the QIDS (one in monitoring, one in CBMv1), and four participants showed reliable deterioration on the GAD7 (two in monitoring, one in CBMv2, one in CBMv3). Possible relatedness of these adverse events to the study was assessed via cross-referencing with these participants’ responses to question 1 on the feedback questionnaire (about whether they felt taking part led to deterioration or improvement in symptoms), and frequency/impact of negative effects on the NEQ that participants attributed to the study, as well as free-text responses in the feedback questionnaire and NEQ. None of these participants reported feeling that the study had led to symptom deterioration on the feedback questionnaire. One participant noted two negative effects on the NEQ: “Unpleasant memories resurfaced” and “I felt that the study did not produce any results”, with impact (how negatively it affected them) for both rated as 1 on the 0 to 4 scale (“Slightly”). No feedback provided in the free text responses on the feedback questionnaire or NEQ for these participants indicated attribution of symptom deterioration to the study. All these adverse events were therefore classified as “Unlikely to be related”. Classification was proposed by SEB and circulated for agreement to the broader study team based on the rationale outlined above.

Tables S5a and S5b shows data from the negative effects questionnaire (NEQ) for the different trial arms. There is no standardised scoring method for the NEQ, and we followed the most recent suggestion of the scale authors (<http://neqscale.com/>) to count the frequency of each event, and the associated impact. The most frequent negative effects that participants attributed to the study were the resurfacing of negative memories, and feeling that the study was not producing any effect.

## Sample Size Comparisons

The sample size comparison presented in Table 3 is based on comparison to the standard way in which psychological treatment development often proceeds, that is, a series of two-arm trials, with a fixed sample size based on a certain level of power at a pre-specified alpha (two-tailed), and adjustment for expected missing data. Power calculations were conducted used the package ‘pwr’ (Champely, 2020), to provide 55% power to find a between-group effect size of *d* = 0.5 at *p* < .09 with 25% missing data (equivalent to the power and false-positive rate for *d* = 0.5 in the trial). This led to a sample size of 36 participants per arm, meaning 36 x 2 x 4 = 288 participants in total for a sequence of 4 two-arm trials.

# References

Blackwell, S. E., Browning, M., Mathews, A., Pictet, A., Welch, J., Davies, J., … Holmes, E. A. (2015). Positive imagery-based cognitive bias modification as a web-based treatment tool for depressed adults: A randomized controlled trial. *Clinical Psychological Science*, *3*(1), 91–111. https://doi.org/10.1177/2167702614560746

Champely, S. (2020). *Pwr: Basic Functions for Power Analysis. R package version 1.3-0.* Retrieved from https://CRAN.R-project.org/package=pwr

Christensen, H., Griffiths, K. M., & Farrer, L. (2009). Adherence in internet interventions for anxiety and depression: Systematic review. *Journal of Medical Internet Research*, *11*(2), e13. https://doi.org/10.2196/jmir.1194

Pictet, A., Jermann, F., & Ceschi, G. (2016). When less could be more: Investigating the effects of a brief internet-based imagery cognitive bias modification intervention in depression. *Behaviour Research and Therapy*, *84*, 45–51. https://doi.org/10.1016/j.brat.2016.07.008

Titov, N., Dear, B. F., Johnston, L., Lorian, C., Zou, J., Wootton, B., … Rapee, R. M. (2013). Improving Adherence and Clinical Outcomes in Self-Guided Internet Treatment for Anxiety and Depression: Randomised Controlled Trial. *PLoS ONE*, *8*(7), e62873. https://doi.org/10.1371/journal.pone.0062873

## *Supplementary Table 1. Detailed Participant Characteristics at Baseline.*

|  | | Monitoring | CBM v1 | CBM v2 | CBM v3 | CBM v4 |
| --- | --- | --- | --- | --- | --- | --- |
|  | | n = 31 | n = 26 | n = 65 | n = 22 | n = 43 |
|  | | M (SD) / N (%) | M (SD) / N (%) | M (SD) / N (%) | M (SD) / N (%) | M (SD) / N (%) |
| Gender | |  |  |  |  |  |
|  | Male | 9 (29.03%) | 7 (26.92%) | 12 (18.75%) | 6 (27.27%) | 9 (20.93%) |
|  | Female | 22 (70.97%) | 19 (73.08%) | 51 (79.69%) | 16 (72.73%) | 34 (79.07%) |
|  | Diverse/Other | 0 (0.00%) | 0 (0.00%) | 1 (1.56%) | 0 (0.00%) | 0 (0.00%) |
| Native German Speaker | |  |  |  |  |  |
|  | Yes | 29 (93.55%) | 23 (88.46%) | 61 (95.31%) | 22 (100.00%) | 40 (93.02%) |
|  | No | 2 (6.45%) | 3 (11.54%) | 3 (4.69%) | 0 (0.00%) | 3 (6.98%) |
| Education level | |  |  |  |  |  |
|  | No school qualifications | 0 (0.00%) | 0 (0.00%) | 0 (0.00%) | 0 (0.00%) | 0 (0.00%) |
|  | High school diploma or equivalent | 11 (35.48%) | 12 (46.15%) | 24 (37.50%) | 9 (40.91%) | 17 (39.53%) |
|  | Vocational college diploma | 9 (29.03%) | 3 (11.54%) | 9 (14.06%) | 5 (22.73%) | 10 (23.26%) |
|  | Bachelor’s degree | 2 (6.45%) | 6 (23.08%) | 9 (14.06%) | 5 (22.73%) | 7 (16.28%) |
|  | Master’s degree | 8 (25.81%) | 3 (11.54%) | 19 (29.69%) | 2 (9.09%) | 6 (13.95%) |
|  | Doctorate /Habilitation | 1 (3.23%) | 2 (7.69%) | 3 (4.69%) | 1 (4.55%) | 3 (6.98%) |
| Occupation | |  |  |  |  |  |
|  | Unemployed / seeking employment | 1 (3.23%) | 3 (11.54%) | 7 (10.94%) | 1 (4.55%) | 3 (6.98%) |
|  | Student / trainee | 6 (19.35%) | 10 (38.46%) | 10 (15.62%) | 5 (22.73%) | 5 (11.63%) |
|  | Self-employed | 3 (9.68%) | 0 (0.00%) | 12 (18.75%) | 3 (13.64%) | 5 (11.63%) |
|  | Employed | 19 (61.29%) | 11 (42.31%) | 31 (48.44%) | 11 (50.00%) | 24 (55.81%) |
|  | Retired | 2 (6.45%) | 2 (7.69%) | 4 (6.25%) | 2 (9.09%) | 6 (13.95%) |
| Current treatment | |  |  |  |  |  |
|  | No | 24 (77.42%) | 19 (73.08%) | 48 (75.00%) | 20 (90.91%) | 34 (79.07%) |
|  | Yes | 7 (22.58%) | 7 (26.92%) | 16 (25.00%) | 2 (9.09%) | 9 (20.93%) |
| Current psychological treatment | |  |  |  |  |  |
|  | No | 26 (83.87%) | 18 (69.23%) | 53 (82.81%) | 19 (86.36%) | 37 (86.05%) |
|  | Yes | 5 (16.13%) | 8 (30.77%) | 11 (17.19%) | 3 (13.64%) | 6 (13.95%) |
| Current medication | |  |  |  |  |  |
|  | No | 26 (83.87%) | 23 (88.46%) | 56 (87.50%) | 20 (90.91%) | 38 (88.37%) |
|  | Yes | 5 (16.13%) | 3 (11.54%) | 8 (12.50%) | 2 (9.09%) | 5 (11.63%) |
| Other current treatment | |  |  |  |  |  |
|  | No | 31 (100.00%) | 26 (100.00%) | 61 (95.31%) | 22 (100.00%) | 41 (95.35%) |
|  | Yes | 0 (0.00%) | 0 (0.00%) | 3 (4.69%) | 0 (0.00%) | 2 (4.65%) |
| Past treatment | |  |  |  |  |  |
|  | No | 14 (45.16%) | 15 (57.69%) | 27 (42.19%) | 14 (63.64%) | 17 (39.53%) |
|  | Yes | 17 (54.84%) | 11 (42.31%) | 37 (57.81%) | 8 (36.36%) | 26 (60.47%) |
| Past psychological treatment | |  |  |  |  |  |
|  | No | 14 (45.16%) | 14 (53.85%) | 32 (50.00%) | 16 (72.73%) | 19 (44.19%) |
|  | Yes | 17 (54.84%) | 12 (46.15%) | 32 (50.00%) | 6 (27.27%) | 24 (55.81%) |
| Past medication | |  |  |  |  |  |
|  | No | 21 (67.74%) | 20 (76.92%) | 41 (64.06%) | 17 (77.27%) | 26 (60.47%) |
|  | Yes | 10 (32.26%) | 6 (23.08%) | 23 (35.94%) | 5 (22.73%) | 17 (39.53%) |
| Past inpatient treatment | |  |  |  |  |  |
|  | No | 27 (87.10%) | 21 (80.77%) | 50 (78.12%) | 19 (86.36%) | 32 (74.42%) |
|  | Yes | 4 (12.90%) | 5 (19.23%) | 14 (21.88%) | 3 (13.64%) | 11 (25.58%) |
| Other past treatment | |  |  |  |  |  |
|  | No | 29 (93.55%) | 26 (100.00%) | 61 (95.31%) | 21 (95.45%) | 41 (95.35%) |
|  | Yes | 2 (6.45%) | 0 (0.00%) | 3 (4.69%) | 1 (4.55%) | 2 (4.65%) |
| Ever received diagnosis? | |  |  |  |  |  |
|  | No | 15 (48.39%) | 13 (50.00%) | 28 (43.75%) | 12 (54.55%) | 17 (39.53%) |
|  | Yes | 16 (51.61%) | 13 (50.00%) | 36 (56.25%) | 10 (45.45%) | 26 (60.47%) |
| Currently a problem? | |  |  |  |  |  |
|  | No | 6 (37.50%) | 4 (30.77%) | 19 (52.78%) | 5 (50.00%) | 13 (50.00%) |
|  | Yes | 10 (62.50%) | 9 (69.23%) | 17 (47.22%) | 5 (50.00%) | 13 (50.00%) |
| Relationship status | |  |  |  |  |  |
|  | Single | 12 (38.71%) | 9 (34.62%) | 27 (42.19%) | 8 (36.36%) | 17 (39.53%) |
|  | Single, in a stable relationship | 5 (16.13%) | 4 (15.38%) | 12 (18.75%) | 4 (18.18%) | 9 (20.93%) |
|  | Married/ Civil partnership / Cohabiting | 12 (38.71%) | 12 (46.15%) | 21 (32.81%) | 7 (31.82%) | 13 (30.23%) |
|  | Divorced / Separated | 1 (3.23%) | 1 (3.85%) | 2 (3.12%) | 3 (13.64%) | 1 (2.33%) |
|  | Widowed | 1 (3.23%) | 0 (0.00%) | 1 (1.56%) | 0 (0.00%) | 2 (4.65%) |
|  | other | 0 (0.00%) | 0 (0.00%) | 1 (1.56%) | 0 (0.00%) | 1 (2.33%) |
| Monthly Household Income | |  |  |  |  |  |
|  | up to 899€ | 6 (19.35%) | 8 (30.77%) | 7 (10.94%) | 6 (27.27%) | 6 (13.95%) |
|  | 900-2099€ | 13 (41.94%) | 5 (19.23%) | 21 (32.81%) | 5 (22.73%) | 11 (25.58%) |
|  | 2100-3099€ | 4 (12.90%) | 4 (15.38%) | 23 (35.94%) | 3 (13.64%) | 9 (20.93%) |
|  | 3100-4099€ | 2 (6.45%) | 3 (11.54%) | 3 (4.69%) | 6 (27.27%) | 11 (25.58%) |
|  | 4100-5099€ | 1 (3.23%) | 4 (15.38%) | 6 (9.38%) | 2 (9.09%) | 5 (11.63%) |
|  | 5100€ or more | 5 (16.13%) | 2 (7.69%) | 4 (6.25%) | 0 (0.00%) | 1 (2.33%) |
| Nationality | |  |  |  |  |  |
|  | German | 26 (83.87%) | 24 (92.31%) | 56 (87.50%) | 21 (95.45%) | 38 (88.37%) |
|  | Turkish | 2 (6.45%) | 2 (7.69%) | 1 (1.56%) | 0 (0.00%) | 1 (2.33%) |
|  | Polish | 0 (0.00%) | 0 (0.00%) | 0 (0.00%) | 0 (0.00%) | 0 (0.00%) |
|  | Italian | 0 (0.00%) | 0 (0.00%) | 0 (0.00%) | 0 (0.00%) | 0 (0.00%) |
|  | Syrian | 0 (0.00%) | 0 (0.00%) | 0 (0.00%) | 0 (0.00%) | 0 (0.00%) |
|  | Romanian | 0 (0.00%) | 0 (0.00%) | 0 (0.00%) | 0 (0.00%) | 0 (0.00%) |
|  | Kurdish | 1 (3.23%) | 0 (0.00%) | 0 (0.00%) | 0 (0.00%) | 0 (0.00%) |
|  | other European | 2 (6.45%) | 0 (0.00%) | 5 (7.81%) | 1 (4.55%) | 4 (9.30%) |
|  | other Asian | 0 (0.00%) | 0 (0.00%) | 1 (1.56%) | 0 (0.00%) | 0 (0.00%) |
|  | other African | 0 (0.00%) | 0 (0.00%) | 0 (0.00%) | 0 (0.00%) | 0 (0.00%) |
|  | other North American | 0 (0.00%) | 0 (0.00%) | 0 (0.00%) | 0 (0.00%) | 0 (0.00%) |
|  | other South American | 0 (0.00%) | 0 (0.00%) | 1 (1.56%) | 0 (0.00%) | 0 (0.00%) |
|  | other Australasian | 0 (0.00%) | 0 (0.00%) | 0 (0.00%) | 0 (0.00%) | 0 (0.00%) |
|  | other Oceania | 0 (0.00%) | 0 (0.00%) | 0 (0.00%) | 0 (0.00%) | 0 (0.00%) |
| Age (years) | | 38.94 (13.29) | 32.85 (10.22) | 41.55 (14.10) | 41.18 (10.99) | 43.49 (14.22) |
| DARS (baseline) | | 64.58 (12.48) | 59.31 (12.51) | 61.69 (14.18) | 63.91 (10.18) | 58.19 (11.48) |
| QIDS (baseline) | | 11.84 (3.64) | 12.58 (4.34) | 12.43 (3.91) | 13.36 (4.15) | 13.21 (4.17) |
| GAD7 (baseline) | | 10.65 (4.90) | 11.31 (4.54) | 11.09 (4.59) | 11.18 (5.05) | 10.58 (4.93) |
| PMH (baseline) | | 12.65 (6.71) | 10.35 (4.27) | 11.74 (5.74) | 9.18 (4.69) | 10.49 (4.43) |
| SUIS (baseline) | | 36.48 (7.33) | 35.00 (8.92) | 38.31 (8.34) | 38.27 (9.31) | 36.07 (7.94) |
| AST (baseline) | | -0.81 (22.03) | -8.77 (16.71) | -5.26 (18.33) | -6.59 (19.88) | -4.67 (17.30) |
| PIT-P (baseline | | 3.30 (0.84) | 3.03 (0.83) | 3.06 (0.91) | 2.91 (0.81) | 2.93 (0.76) |
| PIT-N (baseline) | | 3.29 (0.84) | 3.18 (0.69) | 3.21 (0.89) | 3.40 (0.92) | 3.31 (0.78) |
| CEQ Credibility | | -0.18 (3.04) | -0.41 (2.38) | 0.11 (2.62) | 0.55 (2.40) | -0.07 (2.51) |
| CEQ Expectancy | | 0.22 (3.04) | -0.63 (2.22) | 0.03 (2.66) | -0.18 (2.75) | 0.27 (2.60) |

*Note.* A total of 32 participants were randomized into the Monitoring arm, but one withdrew their data from the trial, hence data for only 31 participants are presented here. CBM v1/2/3/4 = Cognitive Bias Modification version 1/2/3/4. DARS = Dimensional Anhedonia Rating Scale. QIDS = Quick Inventory of Depressive Symptomatology. PMH = Positive Mental Health Scale. SUIS = Spontaneous Use of Imagery Scale. AST = Ambiguous Scenarios Task. PIT-P/N = Prospective Imagery Test – Positive /Negative items. CEQ = Credibility / Expectancy Scale.

## *Table S2. Detailed adherence data in each trial arm, N (%) unless otherwise indicated.*

|  |  | Monitoring | CBM v1 | CBM v2 | CBM v3 | CBM v4 |
| --- | --- | --- | --- | --- | --- | --- |
|  |  | n = 31 | n = 26 | n = 65 | n = 22 | n = 43 |
| Questionnaire adherence | |  |  |  |  |  |
|  | Completed some post-week 1 measures | 25 (80.65%) | 10 (38.46%) | 35 (53.85%) | 18 (81.82%) | 27 (62.79%) |
|  | Completed all post-week 1 measures | 25 (80.65%) | 10 (38.46%) | 35 (53.85%) | 18 (81.82%) | 27 (62.79%) |
|  | Completed some post-week 2 measures | 26 (83.87%) | 6 (23.08%) | 29 (44.62%) | 15 (68.18%) | 24 (55.81%) |
|  | Completed all post-week 2 measures | 26 (83.87%) | 6 (23.08%) | 29 (44.62%) | 15 (68.18%) | 24 (55.81%) |
|  | Completed some post-week 3 measures | 25 (80.65%) | 7 (26.92%) | 23 (35.38%) | 14 (63.64%) | 18 (41.86%) |
|  | Completed all post-week 3 measures | 25 (80.65%) | 7 (26.92%) | 23 (35.38%) | 14 (63.64%) | 17 (39.53%) |
|  | Completed some post-intervention measures | 28 (90.32%) | 9 (34.62%) | 29 (44.62%) | 15 (68.18%) | 21 (48.84%) |
|  | Completed all post-intervention measures | 28 (90.32%) | 9 (34.62%) | 29 (44.62%) | 15 (68.18%) | 20 (46.51%) |
| Training adherence | |  |  |  |  |  |
|  | N training sessions completed, M (SD) |  | 4.65 (4.33) | 5.63 (4.74) | 17.32 (11.63) | 5.23 (4.28) |
|  | % training sessions completed, M (SD) |  | 35.80% (33.28%) | 43.31% (36.43%) | 42.24% (28.36%) | 43.60% (35.63%) |
|  | Started first training session |  | 23 (88.46%) | 58 (89.23%) | 21 (95.45%) | 37 (86.05%) |
|  | Completed first training session |  | 22 (84.62%) | 58 (89.23%) | 20 (90.91%) | 37 (86.05%) |
|  | Completed at least one subsequent session |  | 19 (73.08%) | 47 (72.31%) | 19 (86.36%) | 31 (72.09%) |
|  | Completed all training sessions |  | 3 (11.54%) | 10 (15.38%) | 0 (0.00%) | 7 (16.28%) |
| Mean vividness rating, M (SD) | |  | 4.58 (1.04) | 4.39 (1.09) | 4.56 (1.34) | 4.14 (1.03) |

*Note.*  Participants are only counted here as having started the first training session if they completed at least one training scenario.

## *Figure S1. Observed means and 95% Confidence Intervals for the outcome variables across all trial arms.*

## *Table S3. Outcome data: Observed means and standard deviations, as well as number of participants providing outcome data at each timepoint.*

|  | | Monitoring | CBM v1 | CBM v2 | CBM v3 | CBM v4 |
| --- | --- | --- | --- | --- | --- | --- |
|  | | M (SD, n) | M (SD, n) | M (SD, n) | M (SD, n) | M (SD, n) |
| DARS | |  |  |  |  |  |
|  | Baseline | 64.58 (12.48, 31) | 59.31 (12.51, 26) | 61.69 (14.18, 65) | 63.91 (10.18, 22) | 58.19 (11.48, 43) |
|  | Post-intervention | 65.96 (13.16, 28) | 55.00 (12.19, 9) | 67.34 (15.43, 29) | 68.40 (8.97, 15) | 61.24 (11.23, 21) |
| QIDS | |  |  |  |  |  |
|  | Baseline | 11.84 (3.64, 31) | 12.58 (4.34, 26) | 12.43 (3.91, 65) | 13.36 (4.15, 22) | 13.21 (4.17, 43) |
|  | Post week 1 | 10.32 (3.79, 25) | 8.10 (3.00, 10) | 10.20 (4.70, 35) | 10.00 (5.37, 18) | 8.63 (4.11, 27) |
|  | Post week 2 | 9.19 (4.23, 26) | 7.17 (3.82, 6) | 9.10 (4.60, 29) | 10.07 (5.81, 15) | 8.33 (4.07, 24) |
|  | Post week 3 | 9.20 (5.11, 25) | 8.57 (4.43, 7) | 9.70 (5.57, 23) | 8.29 (4.50, 14) | 7.06 (3.57, 18) |
|  | Post-intervention | 9.07 (4.67, 28) | 8.56 (5.39, 9) | 8.83 (5.61, 29) | 8.27 (5.70, 15) | 8.19 (3.59, 21) |
| GAD7 | |  |  |  |  |  |
|  | Baseline | 10.65 (4.90, 31) | 11.31 (4.54, 26) | 11.09 (4.59, 65) | 11.18 (5.05, 22) | 10.58 (4.93, 43) |
|  | Post week 1 | 10.40 (4.51, 25) | 7.80 (2.66, 10) | 9.49 (5.04, 35) | 10.39 (5.59, 18) | 8.00 (4.19, 27) |
|  | Post week 2 | 9.04 (4.80, 26) | 8.00 (2.76, 6) | 8.21 (4.55, 29) | 9.60 (5.37, 15) | 7.75 (3.61, 24) |
|  | Post week 3 | 10.00 (5.24, 25) | 8.57 (3.31, 7) | 8.87 (5.63, 23) | 9.29 (5.36, 14) | 8.06 (4.16, 17) |
|  | Post-intervention | 9.18 (5.08, 28) | 7.33 (4.18, 9) | 7.86 (4.82, 29) | 8.40 (6.84, 15) | 7.48 (3.66, 21) |
| PMH | |  |  |  |  |  |
|  | Baseline | 12.65 (6.71, 31) | 10.35 (4.27, 26) | 11.74 (5.74, 65) | 9.18 (4.69, 22) | 10.49 (4.43, 43) |
|  | Post week 1 | 12.12 (6.83, 25) | 13.80 (4.76, 10) | 10.94 (5.96, 35) | 9.67 (4.93, 18) | 11.22 (5.67, 27) |
|  | Post week 2 | 12.73 (6.17, 26) | 12.50 (3.27, 6) | 12.45 (6.21, 29) | 9.47 (5.63, 15) | 11.00 (5.88, 24) |
|  | Post week 3 | 11.56 (6.65, 25) | 13.29 (3.15, 7) | 12.30 (6.98, 23) | 10.29 (5.17, 14) | 12.29 (5.71, 17) |
|  | Post-intervention | 13.75 (7.31, 28) | 13.33 (5.94, 9) | 13.72 (7.69, 29) | 11.93 (5.23, 15) | 12.33 (5.40, 21) |
| AST | |  |  |  |  |  |
|  | Baseline | -0.81 (22.03, 31) | -8.77 (16.71, 26) | -5.26 (18.33, 65) | -6.59 (19.88, 22) | -4.67 (17.30, 43) |
|  | Post-intervention | 1.07 (24.87, 28) | -4.22 (26.07, 9) | 6.69 (25.13, 29) | 16.93 (22.35, 15) | 7.50 (22.84, 20) |
| PIT-P | |  |  |  |  |  |
|  | Baseline | 3.30 (0.84, 31) | 3.03 (0.83, 26) | 3.06 (0.91, 65) | 2.91 (0.81, 22) | 2.93 (0.76, 43) |
|  | Post-intervention | 3.34 (1.03, 28) | 3.36 (0.43, 9) | 3.28 (1.04, 29) | 3.36 (0.84, 15) | 3.09 (0.89, 20) |
| PIT-N | |  |  |  |  |  |
|  | Baseline | 3.29 (0.84, 31) | 3.18 (0.69, 26) | 3.21 (0.89, 65) | 3.40 (0.92, 22) | 3.31 (0.78, 43) |
|  | Post-intervention | 3.12 (0.99, 28) | 3.22 (0.57, 9) | 3.25 (0.82, 29) | 3.28 (0.97, 15) | 3.27 (0.82, 20) |

*Note.* CBM v1/2/3/4 = Cognitive Bias Modification version 1/2/3/4. DARS = Dimensional Anhedonia Rating Scale. QIDS = Quick Inventory of Depressive Symptomatology. PMH = Positive Mental Health Scale. AST = Ambiguous Scenarios Task. PIT-P/N = Prospective Imagery Test – Positive /Negative items.

## *Figure S2a.* *Observed means and 95% Confidence Intervals for CBM v1 (n = 26) vs. Monitoring (n = 27).*

## *Figure S2b. Observed means and 95% Confidence Intervals for CBM v2 (n = 29) vs. Monitoring (n = 31).*

## *Figure S2c. Observed means and 95% Confidence Intervals for CBM v3 (n = 22) vs. CBM v2 (n = 18).*

## *Figure S2d. Observed means and 95% Confidence Intervals for CBM v4 (n = 43) vs. CBM v2 (n = 36).*

## *Table S4. Participants responses on the Feedback Questionnaire*

|  | Monitoring | CBM v1 | CBM v2 | CBM v3 | CBM v4 |
| --- | --- | --- | --- | --- | --- |
|  | N = 28 | N = 9 | N = 29 | N = 15 | N = 20 |
| Question | M (SD) | M (SD) | M (SD) | M (SD) | M (SD) |
| 1. To what extent do you believe the study/training made your symptoms better or worse? (-5 = very much worse to +5 = very much better) | 0.43 (1.17) | 0.78 (1.79) | 1.28 (1.75) | 0.67 (2.50) | 1.00 (1.56) |
| 2. How helpful did you find the study/training (1 = not at all, 5 = somewhat, 9 = very) | 3.82 (2.11) | 4.67 (2.45) | 4.93 (2.10) | 5.27 (2.46) | 4.65 (2.76) |
| 3. How did you find the length of the individual sessions? (1 = much too short, 5 = exactly right, 9 = much too long) | 4.46 (1.40) | 7.11 (1.05) | 6.93 (1.69) | 5.40 (1.72) | 6.75 (1.45) |
| 4. How satisfied are you with the study/training? (1 = very unsatisfied, 5 = neither satisfied/dissatisfied, 9 = very satisfied) | 4.96 (1.90) | 5.89 (1.90) | 5.41 (1.72) | 5.00 (2.45) | 5.25 (2.63) |
| 5. How well could you integrate the study/training into your daily life? (1 = not at all feasible, 5 = somewhat feasible, 9 = very feasible) | 7.21 (2.35) | 5.11 (2.47) | 5.28 (2.15) | 5.67 (1.45) | 5.15 (2.76) |
| 6. If a friend was in a similar situation, how confident would you be to recommend the study/training to them? (1 = not at all, 5 = don't know, 9 = very) | 5.11 (2.67) | 4.89 (2.98) | 5.83 (2.04) | 5.60 (2.95) | 5.65 (2.89) |
| 7. If your were in the same situation in future, how motivated would you be to do the study/training again? (1 = not at all, 5 = don't know, 9 = very) | 5.43 (2.86) | 4.44 (3.24) | 5.34 (2.42) | 5.47 (3.40) | 5.80 (3.05) |
| 8. Do you think that the study/training had a positive influence on your: ...Mood? (1 = not at all, 5 = somewhat, 9 = very) | 3.11 (2.25) | 4.11 (2.52) | 4.90 (2.45) | 4.53 (2.77) | 4.20 (2.57) |
| 9. ...Thinking? (1 = not at all, 5 = somewhat, 9 = very) | 3.68 (2.29) | 4.56 (3.09) | 5.21 (2.69) | 5.47 (2.61) | 5.25 (2.83) |
| 10. ...Behaviour? (1 = not at all, 5 = somewhat, 9 = very) | 3.11 (2.44) | 3.56 (2.92) | 4.17 (2.45) | 4.20 (2.65) | 3.95 (2.76) |

## *Table S5a. Participant responses on the Negative Effects Questionnaire (all negative effects).*

|  | | Monitoring | CBM v1 | CBM v2 | CBM v3 | CBM v4 |
| --- | --- | --- | --- | --- | --- | --- |
|  | Question | n = 28 | n = 9 | n = 29 | n = 15 | n = 20 |
| 1. I had more problems with my sleep | | 14 (50.00%) | 3 (33.33%) | 10 (34.48%) | 6 (40.00%) | 4 (20.00%) |
| Impact, M (SD) | | 2.50 (0.94) | 2.00 (1.00) | 2.40 (0.84) | 2.67 (1.03) | 2.25 (1.26) |
| 2. I felt like I was under more stress | | 13 (46.43%) | 6 (66.67%) | 11 (37.93%) | 4 (26.67%) | 5 (25.00%) |
| Impact, M (SD) | | 2.92 (0.76) | 1.67 (0.82) | 1.91 (1.14) | 2.25 (0.50) | 2.80 (0.84) |
| 3. I experienced more anxiety | | 6 (21.43%) | 0 (0.00%) | 2 (6.90%) | 1 (6.67%) | 2 (10.00%) |
| Impact, M (SD) | | 3.33 (0.82) | NA (NA) | 3.00 (1.41) | 1.00 (NA) | 2.00 (1.41) |
| 4. I felt more worried | | 11 (39.29%) | 3 (33.33%) | 8 (27.59%) | 3 (20.00%) | 2 (10.00%) |
| Impact, M (SD) | | 2.36 (1.12) | 1.67 (0.58) | 2.00 (0.93) | 2.00 (1.73) | 3.00 (1.41) |
| 5. I experienced more hopelessness | | 10 (35.71%) | 2 (22.22%) | 8 (27.59%) | 3 (20.00%) | 1 (5.00%) |
| Impact, M (SD) | | 2.40 (1.07) | 1.00 (0.00) | 2.62 (0.74) | 3.33 (0.58) | 4.00 (NA) |
| 6. I experienced more unpleasant feelings | | 9 (32.14%) | 1 (11.11%) | 10 (34.48%) | 6 (40.00%) | 3 (15.00%) |
| Impact, M (SD) | | 2.33 (0.87) | 1.00 (NA) | 1.90 (0.99) | 2.33 (1.21) | 2.67 (0.58) |
| 7. I felt that the issue I was looking for help with got worse | | 2 (7.14%) | 1 (11.11%) | 5 (17.24%) | 3 (20.00%) | 1 (5.00%) |
| Impact, M (SD) | | 3.00 (0.00) | 1.00 (NA) | 2.40 (1.14) | 2.00 (1.00) | 3.00 (NA) |
| 8. Unpleasant memories resurfaced | | 11 (39.29%) | 3 (33.33%) | 17 (58.62%) | 6 (40.00%) | 9 (45.00%) |
| Impact, M (SD) | | 2.09 (1.30) | 1.00 (1.00) | 1.65 (1.06) | 2.00 (0.89) | 1.56 (1.13) |
| 9. I became afraid that other people would find out about my taking part in the study | | 0 (0.00%) | 2 (22.22%) | 1 (3.45%) | 2 (13.33%) | 0 (0.00%) |
| Impact, M (SD) | | NA (NA) | 1.00 (0.00) | 3.00 (NA) | 1.00 (0.00) | NA (NA) |
| 10. I got thoughts that it would be better if I did not exist anymore and that I should take my own life | | 6 (21.43%) | 0 (0.00%) | 7 (24.14%) | 2 (13.33%) | 2 (10.00%) |
| Impact, M (SD) | | 3.00 (0.89) | NA (NA) | 1.57 (1.27) | 2.00 (1.41) | 2.00 (0.00) |
| 11. I started feeling ashamed in front of other people because I was taking part in the study | | 0 (0.00%) | 0 (0.00%) | 2 (6.90%) | 0 (0.00%) | 0 (0.00%) |
| Impact, M (SD) | | NA (NA) | NA (NA) | 2.00 (0.00) | NA (NA) | NA (NA) |
| 12. I stopped thinking that things could get better | | 5 (17.86%) | 1 (11.11%) | 6 (20.69%) | 4 (26.67%) | 1 (5.00%) |
| Impact, M (SD) | | 2.60 (0.55) | 1.00 (NA) | 2.17 (0.75) | 3.50 (0.58) | 4.00 (NA) |
| 13. I started thinking that the issue I was seeking help for could not be made any better | | 7 (25.00%) | 1 (11.11%) | 6 (20.69%) | 5 (33.33%) | 7 (35.00%) |
| Impact, M (SD) | | 2.29 (1.25) | 1.00 (NA) | 1.50 (1.05) | 3.20 (0.45) | 2.57 (1.27) |
| 14. I think that I have developed a dependency on the study | | 2 (7.14%) | 0 (0.00%) | 2 (6.90%) | 0 (0.00%) | 0 (0.00%) |
| Impact, M (SD) | | 0.50 (0.71) | NA (NA) | 1.00 (1.41) | NA (NA) | NA (NA) |
| 15. I did not always understand what I did in the study | | 7 (25.00%) | 1 (11.11%) | 8 (27.59%) | 2 (13.33%) | 2 (10.00%) |
| Impact, M (SD) | | 0.43 (0.53) | 4.00 (NA) | 0.50 (0.76) | 2.00 (2.83) | 1.00 (1.41) |
| 16. I did not always understand the researchers | | 6 (21.43%) | 1 (11.11%) | 5 (17.24%) | 1 (6.67%) | 3 (15.00%) |
| Impact, M (SD) | | 0.67 (0.52) | 4.00 (NA) | 1.00 (0.71) | 4.00 (NA) | 2.33 (2.08) |
| 17. I did not have confidence in the study | | 5 (17.86%) | 3 (33.33%) | 2 (6.90%) | 2 (13.33%) | 4 (20.00%) |
| Impact, M (SD) | | 1.40 (1.52) | 1.67 (2.08) | 0.50 (0.71) | 2.00 (2.83) | 2.75 (0.96) |
| 18. I felt that the study did not produce any results | | 12 (42.86%) | 4 (44.44%) | 12 (41.38%) | 5 (33.33%) | 10 (50.00%) |
| Impact, M (SD) | | 0.83 (1.19) | 1.75 (1.71) | 1.83 (1.19) | 1.60 (1.82) | 1.80 (1.69) |
| 19. I felt that my expectations for the researchers were not fulfilled | | 3 (10.71%) | 1 (11.11%) | 8 (27.59%) | 2 (13.33%) | 5 (25.00%) |
| Impact, M (SD) | | 1.00 (1.00) | 0.00 (NA) | 1.25 (0.89) | 2.00 (2.83) | 1.60 (1.14) |
| 20. I felt that the study was not motivating | | 2 (7.14%) | 2 (22.22%) | 3 (10.34%) | 2 (13.33%) | 4 (20.00%) |
| Impact, M (SD) | | 2.00 (2.83) | 2.00 (2.83) | 1.67 (0.58) | 3.50 (0.71) | 2.50 (1.29) |
| Total number of negative effects, M (SD) | | 4.68 (3.24) | 3.89 (3.02) | 4.59 (3.88) | 3.93 (3.31) | 3.25 (2.97) |

## *Table S5b. Participant responses on the Negative Effects Questionnaire including only those negative effects ascribed to the study*

|  | | Monitoring | CBM v1 | CBM v2 | CBM v3 | CBM v4 |
| --- | --- | --- | --- | --- | --- | --- |
|  | Question | n = 28 | n = 9 | n = 29 | n = 15 | n = 20 |
| 1. I had more problems with my sleep | | 0 (0.00%) | 0 (0.00%) | 1 (3.45%) | 0 (0.00%) | 0 (0.00%) |
| Impact, M (SD) | | NA (NA) | NA (NA) | 2.00 (NA) | NA (NA) | NA (NA) |
| 2. I felt like I was under more stress | | 1 (3.57%) | 1 (11.11%) | 4 (13.79%) | 1 (6.67%) | 2 (10.00%) |
| Impact, M (SD) | | 3.00 (NA) | 1.00 (NA) | 1.25 (0.50) | 2.00 (NA) | 2.00 (0.00) |
| 3. I experienced more anxiety | | 0 (0.00%) | 0 (0.00%) | 0 (0.00%) | 0 (0.00%) | 0 (0.00%) |
| Impact, M (SD) | | NA (NA) | NA (NA) | NA (NA) | NA (NA) | NA (NA) |
| 4. I felt more worried | | 1 (3.57%) | 1 (11.11%) | 3 (10.34%) | 1 (6.67%) | 1 (5.00%) |
| Impact, M (SD) | | 2.00 (NA) | 1.00 (NA) | 1.67 (1.15) | 0.00 (NA) | 2.00 (NA) |
| 5. I experienced more hopelessness | | 2 (7.14%) | 1 (11.11%) | 3 (10.34%) | 1 (6.67%) | 0 (0.00%) |
| Impact, M (SD) | | 1.00 (0.00) | 1.00 (NA) | 2.67 (0.58) | 4.00 (NA) | NA (NA) |
| 6. I experienced more unpleasant feelings | | 1 (3.57%) | 1 (11.11%) | 2 (6.90%) | 1 (6.67%) | 1 (5.00%) |
| Impact, M (SD) | | 2.00 (NA) | 1.00 (NA) | 2.50 (0.71) | 3.00 (NA) | 3.00 (NA) |
| 7. I felt that the issue I was looking for help with got worse | | 0 (0.00%) | 1 (11.11%) | 3 (10.34%) | 2 (13.33%) | 1 (5.00%) |
| Impact, M (SD) | | NA (NA) | 1.00 (NA) | 3.00 (1.00) | 2.00 (1.41) | 3.00 (NA) |
| 8. Unpleasant memories resurfaced | | 5 (17.86%) | 3 (33.33%) | 10 (34.48%) | 3 (20.00%) | 7 (35.00%) |
| Impact, M (SD) | | 1.20 (1.10) | 1.00 (1.00) | 1.20 (0.92) | 2.00 (1.00) | 1.14 (0.69) |
| 9. I became afraid that other people would find out about my taking part in the study | | 0 (0.00%) | 1 (11.11%) | 1 (3.45%) | 2 (13.33%) | 0 (0.00%) |
| Impact, M (SD) | | NA (NA) | 1.00 (NA) | 3.00 (NA) | 1.00 (0.00) | NA (NA) |
| 10. I got thoughts that it would be better if I did not exist anymore and that I should take my own life | | 0 (0.00%) | 0 (0.00%) | 2 (6.90%) | 0 (0.00%) | 0 (0.00%) |
| Impact, M (SD) | | NA (NA) | NA (NA) | 0.50 (0.71) | NA (NA) | NA (NA) |
| 11. I started feeling ashamed in front of other people because I was taking part in the study | | 0 (0.00%) | 0 (0.00%) | 0 (0.00%) | 0 (0.00%) | 0 (0.00%) |
| Impact, M (SD) | | NA (NA) | NA (NA) | NA (NA) | NA (NA) | NA (NA) |
| 12. I stopped thinking that things could get better | | 0 (0.00%) | 1 (11.11%) | 2 (6.90%) | 2 (13.33%) | 0 (0.00%) |
| Impact, M (SD) | | NA (NA) | 1.00 (NA) | 2.50 (0.71) | 3.50 (0.71) | NA (NA) |
| 13. I started thinking that the issue I was seeking help for could not be made any better | | 3 (10.71%) | 1 (11.11%) | 5 (17.24%) | 2 (13.33%) | 5 (25.00%) |
| Impact, M (SD) | | 2.33 (1.53) | 1.00 (NA) | 1.80 (0.84) | 3.50 (0.71) | 2.20 (1.30) |
| 14. I think that I have developed a dependency on the study | | 2 (7.14%) | 0 (0.00%) | 2 (6.90%) | 0 (0.00%) | 0 (0.00%) |
| Impact, M (SD) | | 0.50 (0.71) | NA (NA) | 1.00 (1.41) | NA (NA) | NA (NA) |
| 15. I did not always understand what I did in the study | | 6 (21.43%) | 1 (11.11%) | 6 (20.69%) | 2 (13.33%) | 2 (10.00%) |
| Impact, M (SD) | | 0.50 (0.55) | 4.00 (NA) | 0.67 (0.82) | 2.00 (2.83) | 1.00 (1.41) |
| 16. I did not always understand the researchers | | 6 (21.43%) | 1 (11.11%) | 5 (17.24%) | 1 (6.67%) | 3 (15.00%) |
| Impact, M (SD) | | 0.67 (0.52) | 4.00 (NA) | 1.00 (0.71) | 4.00 (NA) | 2.33 (2.08) |
| 17. I did not have confidence in the study | | 5 (17.86%) | 3 (33.33%) | 2 (6.90%) | 2 (13.33%) | 4 (20.00%) |
| Impact, M (SD) | | 1.40 (1.52) | 1.67 (2.08) | 0.50 (0.71) | 2.00 (2.83) | 2.75 (0.96) |
| 18. I felt that the study did not produce any results | | 12 (42.86%) | 4 (44.44%) | 11 (37.93%) | 5 (33.33%) | 9 (45.00%) |
| Impact, M (SD) | | 0.83 (1.19) | 1.75 (1.71) | 1.91 (1.22) | 1.60 (1.82) | 2.00 (1.66) |
| 19. I felt that my expectations for the researchers were not fulfilled | | 3 (10.71%) | 1 (11.11%) | 8 (27.59%) | 2 (13.33%) | 5 (25.00%) |
| Impact, M (SD) | | 1.00 (1.00) | 0.00 (NA) | 1.25 (0.89) | 2.00 (2.83) | 1.60 (1.14) |
| 20. I felt that the study was not motivating | | 2 (7.14%) | 2 (22.22%) | 3 (10.34%) | 2 (13.33%) | 4 (20.00%) |
| Impact, M (SD) | | 2.00 (2.83) | 2.00 (2.83) | 1.67 (0.58) | 3.50 (0.71) | 2.50 (1.29) |
| Total number of negative effects, M (SD) | | 1.75 (1.90) | 2.56 (2.70) | 2.52 (2.91) | 1.93 (2.76) | 2.20 (2.38) |
